# Switching the reaction pathways of electrochemically generated β-haloalkoxysulfonium ions – synthesis of halohydrins and epoxides

**DOI:** 10.3762/bjoc.11.27

**Published:** 2015-02-13

**Authors:** Akihiro Shimizu, Ryutaro Hayashi, Yosuke Ashikari, Toshiki Nokami, Jun-ichi Yoshida

**Affiliations:** 1 Department of Synthetic Chemistry and Biological Chemistry, Graduate School of Engineering, Kyoto University, Kyotodaigaku-Katsura, Nishikyo-ku, Kyoto 615-8510, Japan

**Keywords:** DMSO, electrosynthesis, epoxides, halohydrins, halogen cations

## Abstract

β-Haloalkoxysulfonium ions generated by the reaction of electrogenerated Br^+^ and I^+^ ions stabilized by dimethyl sulfoxide (DMSO) reacted with sodium hydroxide and sodium methoxide to give the corresponding halohydrins and epoxides, respectively, whereas the treatment with triethylamine gave α-halocarbonyl compounds.

## Introduction

Alkene difunctionalization through three-membered ring halonium ion intermediates [1] is an important transformation in organic synthesis. Usually the halonium ions such as bromonium or iodonium ions are generated by the reaction of alkenes with Br_2_ and I_2_ [2]. However, the most straightforward method is the reaction of alkenes with halogen cations such as Br^+^ and I^+^. The I^+^ cation pool exists as reported by Filimonov et al. [3], although the used solvent is concentrated sulfuric acid which is therefore not compatible with most organic compounds.

**Scheme 1:**
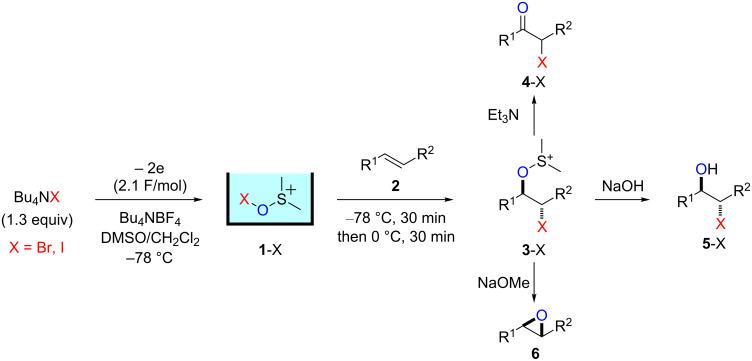
Synthesis of halohydrins and epoxides through β-haloalkoxysulfonium ions generated by the reaction of alkenes with DMSO-stabilized halogen cations.

Electrochemical oxidation [4–11] is a potent technique to generate and accumulate highly reactive cationic species in solution (the “cation pool” method) [12–17]. Although halogen cations are too unstable to accumulate in solution as “cation pools”, halogen cations stabilized by an appropriate stabilizing agent that coordinates the cations can be accumulated in the solution. For example, “I^+^” cations stabilized by acetonitrile (CH_3_CN) [18–20] or by trimethyl orthoformate (TMOF) [21] were reported in the literature. Recently, we reported that dimethyl sulfoxide (DMSO) can also be used to effectively stabilize halogen cations (Scheme 1) [22].

The pools of stabilized halogen cations enable alkene difunctionalization. We previously reported that the reaction of alkenes with DMSO-stabilized halogen cations such as Br^+^ and I^+^ gave β-haloalkoxysulfonium ions and their subsequent treatment with triethylamine gave α-halocarbonyl compounds through Swern–Moffatt-type oxidation [23–27]. Recently reaction integration [28–31] has received significant research interest because it enhances the power and speed of organic syntheses and this is an example of integration of an electrochemical reaction and a chemical reaction using a reactive intermediate. Herein, we report that the reaction pathways of β-haloalkoxysulfonium ions can be switched to give different products by changing the base, thus expanding the utility of the present method. The treatment of β-haloalkoxysulfonium ions **3**-X with sodium hydroxide gave the corresponding halohydrins **5**-X, while the treatment with sodium methoxide gave epoxides **6** (Scheme 1).

## Results and Discussion

### Reactions of β-bromoalkoxysulfonium ions generated from (*Z*)-5-decene

**Scheme 2:**
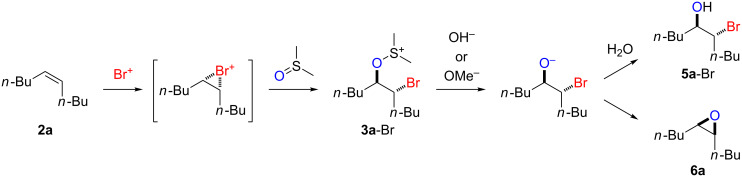
Proposed reaction mechanisms for the syntheses of bromohydrin **5a**-Br and epoxide **6a**.

We first examined the reactions of β-bromoalkoxysulfonium ion **3a**-Br generated by the reaction of (*Z*)-5-decene (**2a**) with Br^+^/DMSO (**1**-Br) [21] (Scheme 1, X = Br). Bu_4_NBr in DMSO/CH_2_Cl_2_ (1:9 v/v) was electrochemically oxidized at −78 °C in a divided cell using Bu_4_NBF_4_ as a supporting electrolyte until 2.1 F/mol of electricity was applied. After addition of **2a** to the solution, the mixture was stirred at 0 °C to give **3a**-Br, which was characterized by NMR spectroscopy [22]. The treatment of **3a**-Br with triethylamine gave α-bromoketone **4a**-Br in 83% yield [22]. However, the treatment of **3a**-Br with NaOH gave bromohydrin **5a**-Br in 89% yield as shown in Table 1. These phenomena can be explained as follows: Due to the steric repulsion, triethylamine cannot attack the sulfur atom in **3a**-Br and acts as base to abstract a proton attached to the carbon adjacent to the sulfur. The formed carbanion part of the resulting sulfur ylide abstracts a proton attached to the carbon adjacent to the oxygen to give α-bromoketone **4a**-Br by the Swern–Moffatt-type oxidation mechanism [23–27]. On the other hand, the hydroxide ion attacks the sulfur atom in **3a**-Br and cleaves the S–O bond to give the alkoxide ion, which is protonated by water to give bromohydrin **5a**-Br (Scheme 2). The stereochemistry determined by NMR (**5a**-Br was synthesized using NBS according to the literature; see Supporting Information File 1) indicated that the addition of Br^+^ and DMSO across the C–C double bond was anti-selective, which is consistent with the results reported previously [22].

**Table 1:** Reaction of **3a**-X (X = Br, I) with different bases.^a^

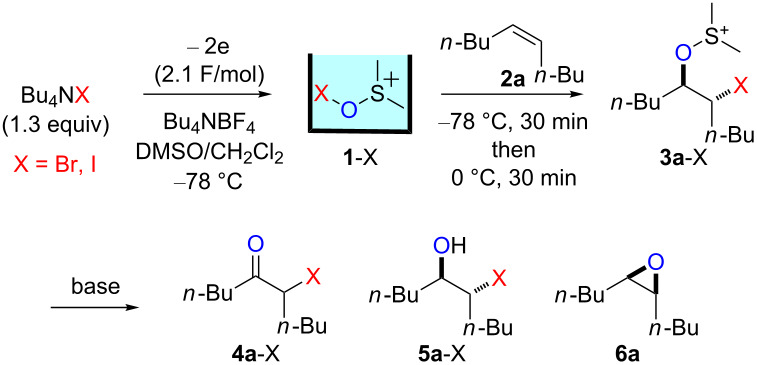							

	% Yield of product^b^						
	X = Br				X = I		
			
Base	**4a**-Br	**5a**-Br	**6a**		**4a**-Ir	**5a**-I	**6a**

Et_3_N/CH_2_Cl_2_	83	ND	ND		85	ND	1
NaOH/H_2_O	ND	89	2		ND	84	1
NaOMe/MeOH	ND	ND	95		ND	ND	96

^a^The electrolysis was carried out using 1.3 equiv of Bu_4_NBr or Bu_4_NI (based on the alkene which was added after electrolysis) with 2.1 F/mol of electricity based on Bu_4_NBr or Bu_4_NI. ^b^Yields were determined by GC.

Treatment of **3a**-Br with NaOMe resulted in a different product, namely epoxide **6a** in 95% yield. In this case, the methoxide ion attacks the sulfur atom and cleaves the S–O bond under formation of an alkoxide ion. The latter intramolecularly attacks the carbon atom bearing the bromine substituent to give epoxide **6a** (Scheme 2). Presumably, the protonation of the alkoxide ion with MeOH is slower than the intramolecular nucleophilic attack. We could not exclude the possibility that a protonated DMSO molecule presumably generated by the reaction of **3a**-Br with the hydroxide ion protonates the alkoxide ion to give **5a**-Br, while a methylated DMSO molecule presumably generated by the reaction of **3a**-Br with the methoxide ion cannot protonate the alkoxide ion, which converts to **6a**. The stereochemistry determined by NMR [32] is consistent with a mechanism involving the back-side attack of the alkoxide ion to form epoxide **6a**.

### Reactions of β-iodoalkoxysulfonium ions generated from (*Z*)-5-decene

We next examined the reactions of β-iodoalkoxysulfonium ion **3a**-I generated by the reaction of (*Z*)-5-decene (**2a**) with I^+^/DMSO (**1**-I) cation pool [22] (Scheme 1, X = I). Bu_4_NI in DMSO/CH_2_Cl_2_ (1:9 v/v) was electrochemically oxidized at −78 °C in a divided cell using Bu_4_NBF_4_ as a supporting electrolyte until 2.1 F/mol of electricity was applied. After addition of **2a** to the solution, the mixture was stirred at 0 °C to give **3a**-I, which was characterized by NMR spectroscopy [22]. The treatment of **3a**-I with triethylamine gave α-iodoketone **4a**-I in 85% yield as we reported previously [22]. However, the treatment of **3a**-I with NaOH and NaOMe gave iodohydrin **5a**-I in 84% yield and epoxide **6a** in 96% yield, respectively (Table 1). The stereochemistry as determined by NMR (**5a**-I was synthesized using I_2_ and H_2_O_2_; see Supporting Information File 1) indicated that the addition of I^+^ and DMSO across the C–C double bond was anti-selective as anticipated.

### Synthesis of halohydrins and epoxides from various alkenes

The present method was successfully applied to the synthesis of halohydrins and epoxides from various alkenes. The reactions of alkenes with **1**-X followed by the treatment with NaOH gave the corresponding halohydrins as shown in Table 2. The reactions of *E* and *Z* isomers of 1-phenyl-1-propene (**2d**) with **1**-Br gave **5d**-Br and **5d’**-Br, respectively (Table 2, entries 7 and 9), indicating the anti-addition of Br^+^ and DMSO across the C–C double bond. The reaction with **1**-I also gave the anti-addition products (Table 2, entries 8 and 10). Therefore, the reaction is stereospecific, and the stereochemistry is consistent with the proposed reaction mechanism (Scheme 2). The addition of Br^+^ or I^+^ and DMSO to unsymmetrically substituted olefins **2c** and **2d** regioselectively gave bromohydrins as single regioisomers (Table 2, entries 5–10). The regioselectivity of the products can be explained by the stability of carbocations (benzyl > secondary > primary). In the case of terminal alkene **2c**, Br and I were introduced to a primary carbon atom, whereas OH was introduced to a secondary carbon atom. In the case of styrene derivative **2d**, Br and I were introduced to a secondary carbon, whereas OH was introduced to the benzyl carbon. DMSO seems to attack the more positively charged carbon of the three-membered ring bromonium ion or iodonium ion.

**Table 2:** Synthesis of halohydrins by the reaction of **1**-X with alkenes followed by the treatment with NaOH.^a^

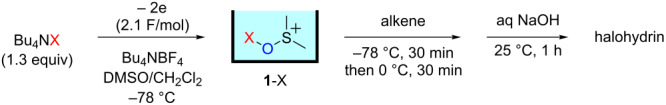			

Entry	Alkene	Product	Yield (%)^b^

1	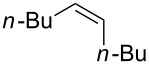 **2a**	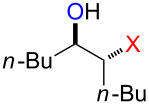 **5a**-Br, **5a**-I	**5a**-Br: 87
2			**5a**-I: 84^c^
3	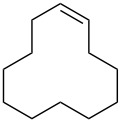 **2b** (*Z*:*E* = 72:28)	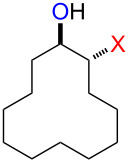 **5b**-Br, **5b**-I	**5b**-Br: 74 (trans:cis = 79:21)
4			**5b**-I: 94 (trans:cis = 71:29)
5	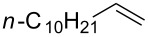 **2c**	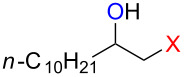 **5c**-Br, **5c**-I	**5c**-Br: 57
6			**5c**-I: 53
7	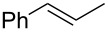 (*E*)-**2d**	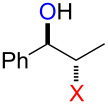 **5d**-Br, **5d**-I	**5d**-Br: 73
8			**5d**-I: 35
9	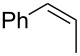 (*Z*)-**2d**	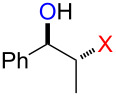 **5d’**-Br, **5d’**-I	**5d’**-Br: 75
10			**5d’**-I: 51

^a^The electrolysis of Bu_4_NBr and Bu_4_NI was carried out using 1.3 equiv of Bu_4_NX (based on the alkene which was added after the electrolysis) with 2.1 F/mol of electricity based on Bu_4_NX. ^b^Isolated yield. ^c^Yield was determined by GC.

The reaction of **1**-X with alkenes followed by the treatment with NaOMe gave the corresponding epoxides as shown in Table 3. Alkenes having an alkoxycarbonyl group gave the corresponding epoxides in moderate yields (Table 3, entries 11–14). Diene **2f** reacted with **1**-Br and **1**-I to give monoepoxide **6f** in moderate yields (Table 3, entries 13 and 14). Interestingly, **2g** reacted with **1**-Br to give **6g** but not with **1**-I (Table 3, entries 15 and 16), although the reason is not clear at present. The facial selectivity of the reaction is the opposite to that of the epoxidation using conventional reagents such as *m*-chloroperoxybenzoic acid (mCPBA) which epoxidizes alkenes from the less hindered face [33–34]. In this reaction, Br^+^ adds to the C–C double bond of **2g** from the less hindered face to form the corresponding three-membered ring bromonium ion intermediate. Subsequently, DMSO attacks the bromonium ion from the more hindered face to form the corresponding β-haloalkoxysulfonium ion. The treatment of the β-haloalkoxysulfonium ion with NaOMe cleaves the O–S bond to generate the alkoxide ion, which attacks the carbon atom bearing bromine to give epoxide **6g**. Therefore, the installation of the oxygen atom takes place from the more hindered face.

**Table 3:** Synthesis of epoxides by the reaction of **1**-X with alkenes followed by the treatment with NaOMe.^a^

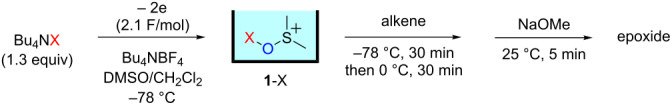				

Entry	Alkene	Product	X	Yield (%)^b^

1	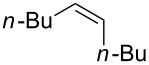 **2a**	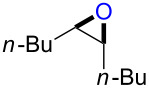 **6a**	Br	95^c^
2			I	96^c^
3	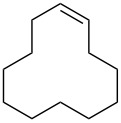 **2b** (*Z*:*E* = 72:28)	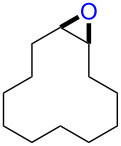 **6b**	Br	68 (*cis*:*trans* = 74:26)
4			I	89 (*cis*:*trans* = 74:26)
5	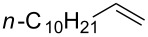 **2c**	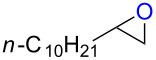 **6c**	Br	73^c^
6			I	86^c^
7	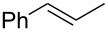 (*E*)-**2d**	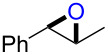 **6d**	Br	53
8			I	38^d^
9	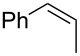 (*Z*)-**2d**	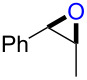 **6d’**	Br	60
10			I	67^d^
11	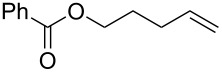 **2e**	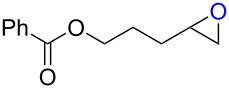 **6e**	Br	52^e^
12			I	57^e^
13	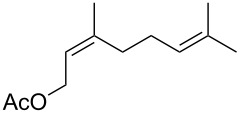 **2f**	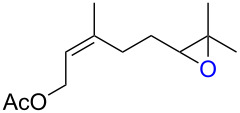 **6f**	Br	49^e^
14			I	47^e^
15	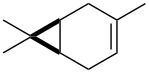 **2g**	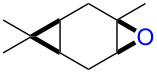 **6g**	Br	69
16			I	0

^a^The electrolysis was carried out using 1.3 equiv of Bu_4_NBr or Bu_4_NI (based on the alkene which was added after electrolysis) with 2.1 F/mol of electricity based on Bu_4_NBr or Bu_4_NI. ^b^Isolated yield. ^c^Yield was determined by GC. ^d^2.0 Equiv of Bu_4_NI was used. ^e^Reacted with 2.5 equiv of NaOMe for 2 h.

### Reaction mechanism

**Scheme 3:**
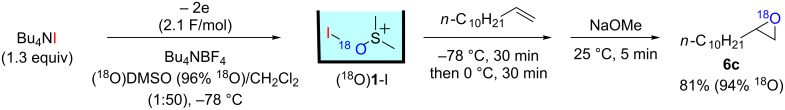
Mechanistic study using ^18^O-DMSO.

To confirm the mechanism shown in Scheme 2, the experiment was repeated using ^18^O-labeled DMSO (96% ^18^O)/CH_2_Cl_2_ (1:50 v/v). As outlined in Scheme 3, epoxide **6c** containing ^18^O (94% ^18^O) was obtained in 81% yield, indicating that the oxygen atom in the product originated from DMSO. Since ^18^O-labeled DMSO can be easily obtained from H_2_^18^O [35], the present transformation serves as a convenient method for synthesizing ^18^O-labeled epoxides, that can be used for various mechanistic and biological studies.

## Conclusion

In conclusion, we found that the reaction pathways of β-haloalkoxysulfonium ions generated by the reaction of electrogenerated Br^+^ and I^+^ stabilized by dimethyl sulfoxide (DMSO) can be switched by changing the nature of the base. The present transformation serves as stereospecific route to halohydrins and epoxides from alkenes. The method is also useful for synthesizing ^18^O-labeled epoxides.

## Supporting Information

File 1 Experimental and analytical data.
