# Supplementary material for: Switching the reaction pathways of electrochemically generated β-haloalkoxysulfonium ions – synthesis of halohydrins and epoxides
Source: Beilstein J Org Chem. 2015 Feb 13;11:242–8. doi: 10.3762/bjoc.11.27 (PMC4362321; doi:10.3762/bjoc.11.27)
Supplement: File 1 — Experimental and analytical data. [file Beilstein_J_Org_Chem-11-242-s001.pdf]

Supporting Information

for

Switching the reaction pathways of electrochemically generated β-haloalkoxysulfonium ions – synthesis of halohydrins and epoxides

Akihiro Shimizu, Ryutaro Hayashi, Yosuke Ashikari, Toshiki Nokami[§], and Jun-ichi Yoshida*

Address: Department of Synthetic Chemistry and Biological Chemistry, Graduate School of Engineering, Kyoto University, Kyotodaigaku-Katsura, Nishikyo-ku, Kyoto 615-8510, Japan

Email: Jun-ichi Yoshida - yoshida@sbchem.kyoto-u.ac.jp

*Corresponding author

[§]Present address: Department of Chemistry and Biotechnology, Graduate School of Engineering, Tottori University, 4-101 Koyamachominami, Tottori 680-8552, Japan

Experimental and analytical data

Table of Contents

1. General S2
2. Reaction of **3a**-X (X = Br, I) with bases..................................... S2–S4
3. Synthesis of halohydrins..................................... S4–S9
4. Synthesis of epoxides S9–S14
5. Synthesis of ^{18}O-labelled epoxide S15
6. References S16
7. NMR spectra..................................... S17–S46
8. Mass spectra of ^{18}O-labelled DMSO and **6c**..................................... S47–S48

1. General

^{1}H and ^{13}C NMR spectra were recorded in $CDCl_3$ using a Varian MERCURY plus-400 (^{1}H 400 MHz, ^{13}C 100 MHz) spectrometer, or a JEOL ECA-600P spectrometer (^{1}H 600 MHz, ^{13}C 150 MHz) with tetramethylsilane as an internal standard unless otherwise noted. Mass spectra were obtained on JEOL EXACTIVE (ESI and APCI) mass spectrometer, and JEOL JMS-SX102A mass spectrometer (EI). GC analysis was performed on a SHIMADZU GC-2014 gas chromatograph equipped with a flame ionization detector using a fused silica capillary column (column, CBP1; 0.22 mm x 25 m). Merck precoated silica gel F254 plates (thickness 0.25 mm) were used for thin layer chromatography (TLC) analysis. Flash chromatography was carried out on a silica gel (Kanto Chem. Co., Silica Gel N, spherical, neutral, 40–100 μm) unless otherwise noted. All reactions were carried out under argon atmosphere unless otherwise noted. The anodic oxidation was carried out using an H-type divided cell (4G glass filter) equipped with a carbon felt anode (Nippon Carbon GF-20-P21E, ca. 160 mg for 0.25 mmol scale, dried at 300 °C/1 mmHg for 3 h before use) and a platinum plate cathode (10 mm x 10 mm). Bu_4NBF_4 was purchased from TCI and dried at 25 °C/1 mmHg for 12 h. Dichloromethane was washed with water, distilled from P_2O_5, redistilled from dry K_2CO_3 to remove trace amounts of acid, and stored over molecular sieves 4 Å. Dimethyl sulfoxide (DMSO) and triethylamine were dried over molecular sieves 4 Å before use. Unless otherwise noted, all materials were obtained from commercial suppliers and used without further purification.

2. Reaction of 3a-X (X = Br, I) with bases

Generation of **1**-Br and **1**-I was conducted in a similar manner to a procedure from reference[1].

Typical procedure for generation of 1-Br and 3a-Br and reaction of 3a-Br with bases

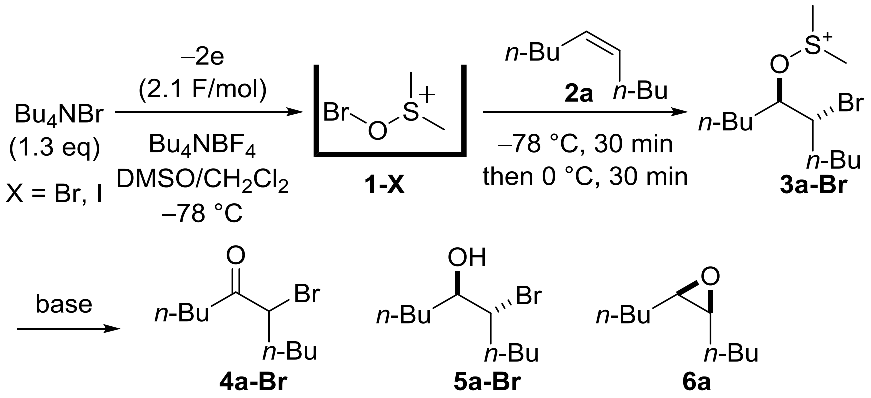

In the anodic chamber were placed Bu_4NBr (80.7 mg, 0.251 mmol), Bu_4NBF_4 (100 mg, 0.3 mmol), DMSO (1 mL), and 0.3 M Bu_4NBF_4/CH_2Cl_2 (9 mL). In the cathodic chamber were placed TfOH (60 μL, 0.68 mmol) and 0.3 M Bu_4NBF_4/CH_2Cl_2 (10 mL). The constant current electrolysis (8.0 mA) was carried out at −78 °C with magnetic stirring until 2.1 F mol^{-1} of electricity was consumed. To the anodic chamber was added a solution of (*Z*)-5-decene (**2a**) (27.7 mg, 0.197 mmol) in CH_2Cl_2 (0.5 mL), and to the cathodic chamber 0.5 mL of CH_2Cl_2 was added at −78 °C. The solution was stirred for 30 min at −78 °C then stirring was stirred continued for 30 min at 0 °C. Et_3N (100 μL) was added to both the anodic and the cathodic chambers, and the resulting mixture was warmed to 25 °C and stirred for 1 h. The solution in the anodic

chamber was collected and the solvent was removed under reduced pressure. The residue was filtered through a short column (2 x 4 cm) of silica gel to remove Bu_4NBF_4 by using hexane/EtOAc (1:1) as an eluent. The GC analysis using hexadecane as an internal standard indicated that 6-bromodecan-5-one (**4a**-Br) was obtained in 83% yield (38.6 mg, 0.164 mmol). The ^{1}H NMR data was reported previously[1]. Addition of NaOH (2.5 M in H_2O, 0.16 mL) instead of Et_3N gave (5*R**,6*R**)-6-bromodecan-5-ol (**5a**-Br) in 89% yield (0.174 mmol). TLC R_f 0.19 (hexane/EtOAc 20:1); ^{1}H NMR (400 MHz, $CDCl_3$) δ 0.92 (t, *J* = 7.2 Hz, 6 H), 1.25−1.61 (m, 10 H), 1.84 (d, *J* = 8.0 Hz, 1 H), 1.86−2.00 (m, 2 H), 3.20−3.57 (m, 1 H), 4.07 (ddd, *J* = 3.2, 5.2, 8.4 Hz, 1 H); The ^{1}H NMR spectrum is in agreement with that of an authentic sample synthesized using NBS according to the literature[2] (*vide infra*). Addition of NaOMe (5.0 M in MeOH, 0.2 mL) instead of Et_3N gave **(5*R**,6*S**)-5,6-epoxydecane** (**6a**) in 95% yield (0.187 mmol). TLC R_f 0.83 (hexane/EtOAc 5:1); ^{1}H NMR (400 MHz, $CDCl_3$) δ 0.93 (t, *J* = 7.2 Hz, 6 H), 1.37−1.55 (m, 12 H), 2.88−2.94 (m, 2 H); ^{13}C NMR (100 MHz, $CDCl_3$) δ 14.0, 22.6, 27.5, 28.3, 57.2; HRMS (APCI) calcd for $C_{10}H_{21}O$ $(M+H^+)$: 157.1587, found: 157.1587. Stereochemistry was determined by comparison of the ^{1}H NMR spectrum with that in the literature[3].

Typical procedure for the generation of 1-I and 3a-I and reaction of 3a-I with bases

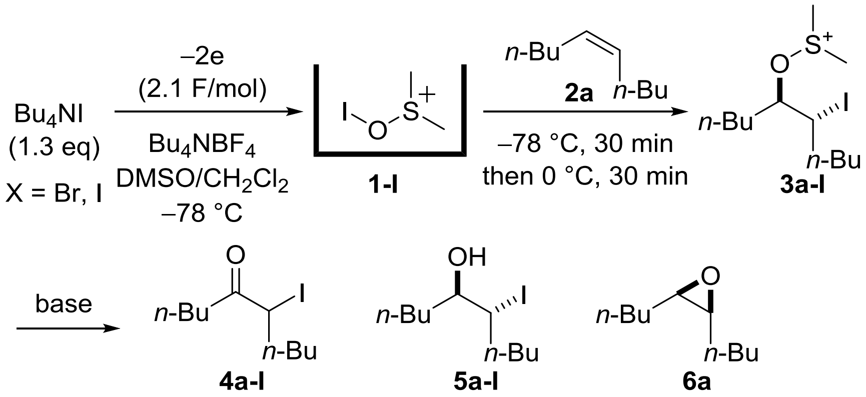

In the anodic chamber were placed Bu_4NI (91.6 mg, 0.248 mmol), Bu_4NBF_4 (102 mg, 0.3 mmol), DMSO (1 mL), and 0.3 M Bu_4NBF_4/CH_2Cl_2 (9 mL). In the cathodic chamber were placed TfOH (60 μL, 0.68 mmol) and 0.3 M Bu_4NBF_4/CH_2Cl_2 (10 mL). The constant current electrolysis (8.0 mA) was carried out at −78 °C with magnetic stirring until 2.1 F mol^{-1} of electricity was consumed. To the anodic chamber was added a solution of (*Z*)-5-decene (**2a**) (28.7 mg, 0.205 mmol) in CH_2Cl_2 (0.5 mL), and to the cathodic chamber was added 0.5 mL of CH_2Cl_2 at −78 °C. The solution was stirred for 30 min at −78 °C then stirring was continued for 30 min at 0 °C. Et_3N (100 μL) was added to both the anodic and the cathodic chambers, and the resulting mixture was warmed to 25 °C and stirred for 1 h. The solution in the anodic chamber was collected and the solvent was removed under reduced pressure. The residue was filtered through a short column (2 x 4 cm) of silica gel to remove Bu_4NBF_4 by using hexane/EtOAc (1:1 v/v) as an eluent. The GC analysis using hexadecane as an internal standard indicated that 6-iododecan-5-one (**4a**-I) was obtained in 85% yield (49.2 mg, 0.174 mmol). The ^{1}H NMR data was reported previously[1]. Addition of NaOH (2.5 M in H_2O, 0.16 mL) instead of Et_3N gave (5*R**,6*R**)-6-iododecan-5-ol (**5a**-I) in 84% yield (0.169 mmol). TLC R_f 0.19 (hexane/EtOAc 20:1); ^{1}H NMR (400 MHz, $CDCl_3$) δ 0.92 (t, *J* = 7.2 Hz, 6 H), 1.25−1.61 (m, 10 H), 1.84 (d,

J = 8.0 Hz, 1 H), 1.86–2.00 (m, 2 H), 3.20–3.57 (m, 1 H), 4.07 (ddd, J = 3.2, 5.2, 8.4 Hz, 1 H); The ^{1}H NMR spectrum is in agreement with that of an authentic sample synthesized using I_2 and H_2O_2 according to the literature[4] (*vide infra*). Addition of NaOMe (5.0 M in MeOH, 0.2 mL) instead of Et_3N gave (5*R**,6*S**)-5,6-epoxydecane (**6a**) in 96% yield (0.191 mmol). TLC R_f 0.83 (hexane/EtOAc 5:1); ^{1}H NMR (400 MHz, $CDCl_3$) δ 0.93 (t, J = 7.2 Hz, 6 H), 1.37–1.55 (m, 12 H), 2.88–2.94 (m, 2 H); ^{13}C NMR (100 MHz, $CDCl_3$) δ 14.0, 22.6, 27.5, 28.3, 57.2; HRMS (APCI) calcd for $C_{10}H_{21}O$ (M+H$^+$): 157.1587, found: 157.1587. Stereochemistry was determined by comparison of the ^{1}H NMR spectrum with that in the literature[3].

3. Synthesis of halohydrins

Generation of **1**-Br and **1**-I was conducted in a similar manner to a procedure from ref. 1.

Typical procedure for generation of 1-Br and synthesis of bromohydrins

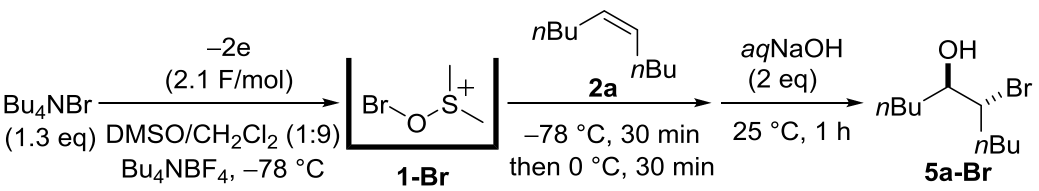

In the anodic chamber were placed Bu_4NBr (81.0 mg, 0.252 mmol), Bu_4NBF_4 (101 mg, 0.3 mmol), DMSO (1 mL), and 0.3 M Bu_4NBF_4/CH_2Cl_2 (9 mL). In the cathodic chamber were placed TfOH (60 μL, 0.68 mmol) and 0.3 M Bu_4NBF_4/CH_2Cl_2 (10 mL). The constant current electrolysis (8.0 mA) was carried out at −78 °C with magnetic stirring until 2.1 F mol^{-1} of electricity was consumed. To the anodic chamber was added a solution of (*Z*)-5-decene (**2a**) (27.0 mg, 0.193 mmol) in CH_2Cl_2 (0.5 mL), and to the cathodic chamber 0.5 mL of CH_2Cl_2 was added at −78 °C. The solution was stirred for 30 min at −78 °C then stirring was continued for 30 min at 0 °C. NaOH (2.5 M in H_2O, 0.16 mL) was added to both the anodic and the cathodic chambers, and the resulting mixture was warmed to 25 °C and was stirred for 1 h. The solution in the anodic chamber was collected and the solvent was removed under reduced pressure. The residue was filtered through a short column (2 x 4 cm) of silica gel to remove Bu_4NBF_4 by using hexane/EtOAc (1:1) as an eluent. After removal of the solvent under reduced pressure, the crude product was purified by flash chromatography (hexane/EtOAc 20:1) to obtain (5*R**,6*R**)-6-bromodecan-5-ol (**5a**-Br) in 87% yield (40.0 mg, 0.169 mmol). TLC R_f 0.19 (hexane/EtOAc 20:1); ^{1}H NMR (400 MHz, $CDCl_3$) δ 0.92 (t, J = 7.2 Hz, 6 H), 1.25–1.61 (m, 10 H), 1.84 (d, J = 8.0 Hz, 1 H), 1.86–2.00 (m, 2 H), 3.20–3.57 (m, 1 H), 4.07 (ddd, J = 3.2, 5.2, 8.4 Hz, 1 H); The ^{1}H NMR spectrum is in agreement with that of an authentic sample synthesized using NBS according to the literature[2] (*vide infra*) .

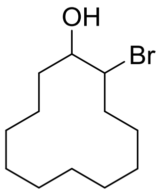

5b-Br

2-Bromocyclododecan-1-ol (5b-Br). Electrochemical oxidation (2.1 F mol^{-1}) of Bu_4NBr (82 mg, 0.25 mmol), subsequent addition of the solution of cyclododecene (**2b**) (*Z*/*E* = 72:28, 31.6 mg, 0.190 mmol) in CH_2Cl_2 (0.5 mL), and the treatment with NaOH followed by flash chromatography (hexane/EtOAc 20:1) gave the title compound as mixture of the diastereomers (37.3 mg, 0.142mmol, 74%, *trans:cis* = 79:21). TLC R_f 0.42 and 0.48 (hexane/EtOAc 5:1); ^1H NMR (400 MHz, $CDCl_3$) δ 1.24−1.82 (m, 18 H), 1.85−1.96 (m, 1 H), 2.01−2.16 (m, 2 H), 2.23 (d, *J* = 5.2 Hz, 1 H), 3.80 (td, *J* = 5.2, 12.0 Hz, trans 1 H), 3.90 (br, cis 1 H), 4.32−4.41 (m, 1 H); The ^1H NMR spectrum is in agreement with that in the literature[5].

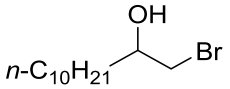

5c-Br

1-Bromododecan-2-ol (5c-Br). Electrochemical oxidation (2.1 F mol^{-1}) of Bu_4NBr (81 mg, 0.25 mmol), subsequent addition of the solution of 1-dodecene (**2c**) (32.5 mg, 0.193 mmol) in CH_2Cl_2 (0.5 mL), and the treatment with NaOH followed by flash chromatography (hexane/EtOAc 100:0 to 50:1) gave the title compound (29.3 mg, 0.110 mmol, 57%). TLC R_f 0.48 (hexane/EtOAc 5:1); ^1H NMR (400 MHz, $CDCl_3$) δ 0.88 (t, *J* = 6.8 Hz, 3 H), 1.23−1.61 (m, 20 H), 2.10−2.14 (m, 1 H), 3.39 (dd, *J* = 7.2, 10.4 Hz, 1 H), 3.55 (dd, *J* = 2.8, 10.4 Hz, 1 H), 3.74−3.82 (m, 1 H); ^{13}C NMR (100 MHz, $CDCl_3$) δ 14.1, 22.7, 25.6, 29.3, 29.47, 29.48, 29.54, 29.6, 31.9, 35.1, 40.7, 71.1; HRMS (EI) calcd for $C_{12}H_{24}OBr$ ($M-H^+$): 263.1016, found: 263.1013. The ^1H NMR spectrum is in agreement with that in the literature[6].

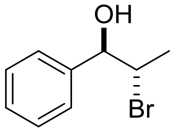

5d-Br

(1*R,2*S**)-2-Bromo-1-phenyl-1-propanol** (**5d-Br**). Electrochemical oxidation (2.1 F mol^{-1}) of Bu_4NBr (81.2 mg, 0.252 mmol), subsequent addition of the solution of (*E*)-β-methylstylene ((***E***)**-2d**) (22.8 mg, 0.193 mmol) in CH_2Cl_2 (0.5 mL), and the treatment with NaOH followed by flash chromatography (hexane/EtOAc 100:0 to 20:1) gave the title compound (30.2 mg, 0.140 mmol, 73%). TLC R_f 0.33 (hexane/EtOAc 5:1); ^1H NMR (400 MHz, $CDCl_3$) δ 1.54 (d, *J* = 6.8 Hz, 3 H), 2.51 (m, 1 H), 4.43 (dq, *J* = 3.6, 6.8 Hz, 1 H), 5.01 (t, *J* = 3.6 Hz, 1 H), 7.27–7.40 (m, 5 H). The ^1H NMR spectrum is in agreement with that in the literature[1].

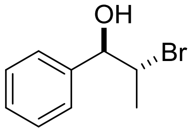

5d'-Br

(1*R,2*R**)-2-Bromo-1-phenyl-1-propanol** (**5d’-Br**)**.** Electrochemical oxidation (2.1 F mol^{-1}) of Bu_4NBr (81.9 mg, 0.254 mmol), subsequent addition of the solution of (*Z*)-β-methylstylene ((***Z***)**-2d**) (23.5 mg, 0.199 mmol) in CH_2Cl_2 (0.5 mL), and the treatment with NaOH followed by flash chromatography (hexane/EtOAc 100:0 to 20:1) gave the title compound (32.2 mg, 0.150 mmol, 75%). TLC R_f 0.38 (hexane/EtOAc 5:1); ^1H NMR (400 MHz, $CDCl_3$) δ 1.57 (d, *J* = 6.8 Hz, 3 H), 2.77 (m, 1 H), 4.34 (dq, *J* = 6.8, 7.6 Hz, 1 H), 4.62 (dd, *J* = 3.6, 7.6 Hz, 1 H), 7.30–7.40 (m, 5 H). The ^1H NMR spectrum is in agreement with that in the literature[1].

Synthesis of (5*R,6*R**)-6-bromodecan-5-ol (5a-Br)**

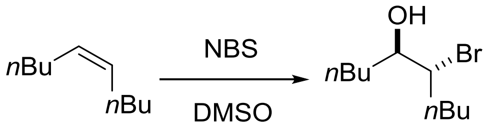

To a round-bottom flask were added NBS (640 mg, 3.59 mmol), (*Z*)-5-decene (417 mg, 2.97 mmol), DMSO (10 mL), and H_2O (0.1 mL) and the mixture was stirred for 4 h. After the addition of NBS (600 mg, 3.37 mmol), the mixture was stirred at room temperature for 1 d. The solution was diluted with EtOAc (30 mL), washed with sat aq $NaHCO_3$ (10 mL x 2), H_2O (10 mL x 2), and brine (10 mL) and dried over Na_2SO_4. After removal of the solvent under reduced pressure, the crude product was purified by flash chromatography (hexane/EtOAc 100:0 to 20:1) to obtain (5*R**,6*R**)-6-bromodecan-5-ol (**5a**-Br) in 60% yield (420 mg, 1.77 mmol). TLC R_f 0.19 (hexane/EtOAc 20:1); ^{1}H NMR (400 MHz, $CDCl_3$) δ 0.92 (t, *J* = 7.2 Hz, 6 H), 1.25−1.61 (m, 10 H), 1.84 (d, *J* = 8.0 Hz, 1 H), 1.86−2.00 (m, 2 H), 3.20−3.57 (m, 1 H), 4.07 (ddd, *J* = 3.2, 5.2, 8.4 Hz, 1 H); ^{13}C NMR (100 MHz, $CDCl_3$) δ 13.9, 14.0, 22.1, 22.6, 27.8, 30.0, 35.48, 35.52, 65.5, 73.8; HRMS (EI) calcd for $C_{10}H_{20}OBr$ ($M-H^+$): 235.0703, found: 235.0702.

Typical procedure for the generation of 1-I and the synthesis of iodohydrins

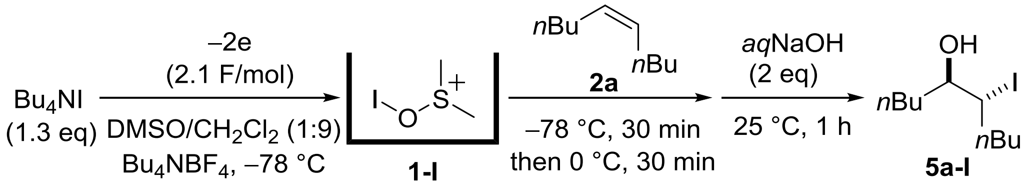

In the anodic chamber were placed Bu_4NI (93 mg, 0.25 mmol), Bu_4NBF_4 (980 mg, 3.0 mmol), DMSO (1 mL), and CH_2Cl_2 (9 mL). In the cathodic chamber were placed TfOH (60 μL, 0.68 mmol) and 0.3 M Bu_4NBF_4/CH_2Cl_2 (10 mL). The constant current electrolysis (8.0 mA) was carried out at −78 °C with magnetic stirring until 2.1 F mol^{-1} of electricity was consumed. To the anodic chamber was added a solution of (*Z*)-5-decene (**2a**) (27.0 mg, 0.193 mmol) in CH_2Cl_2 (0.5 mL), and to the cathodic chamber was added 0.5 mL of CH_2Cl_2 at −78 °C. The solution was stirred for 30 min at −78 °C then stirring was continued for 30 min at 0 °C. NaOH (2.5 M in H_2O, 0.16 mL) was added to both the anodic and the cathodic chambers, and the resulting mixture was warmed to 25 °C and stirred for 1 h. The solution in the anodic chamber was collected and the solvent was removed under reduced pressure. The residue was filtered through a short column (2 x 4 cm) of silica gel to remove Bu_4NBF_4 by using hexane/EtOAc (1:1 v/v) as an eluent. The GC analysis using hexadecane as an internal standard indicated that (5*R**,6*R**)-6-iododecan-5-ol (**5a**-I) was obtained in 84% yield (0.169 mmol). TLC R_f 0.56 (hexane/EtOAc 5:1); ^{1}H NMR (400 MHz, $CDCl_3$) δ 0.92 (t, *J* = 7.2 Hz, 6 H), 1.25–1.46 (m, 6 H), 1.47–1.60 (m, 4 H), 1.62 (d, *J* = 8.4 Hz, 1 H), 1.78–1.88 (m, 1 H), 1.98–2.09 (m, 1 H), 2.85–2.90 (m, 1 H), 4.19 (ddd, *J* = 2.8, 4.8, 9.2 Hz, 1 H); The ^{1}H NMR spectrum is in agreement with that of an authentic sample synthesized using I_2 and H_2O_2 according to the literature[4] (*vide infra*).

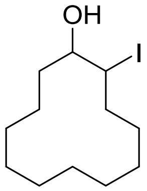

5b-I

2-Iodocyclododecan-1-ol (5b-I). Electrochemical oxidation (2.1 F mol^{-1}) of Bu_4NI (90.8mg, 0.246 mmol), subsequent addition of the solution of cyclododecene (**2b**) (*Z*/*E* = 72:28, 31.4 mg, 0.189 mmol) in CH_2Cl_2 (0.5 mL), and the treatment with NaOH followed by flash chromatography (hexane/EtOAc 20:1) gave the title compound (55.0 mg, 0.177 mmol, 94%, *trans:cis* = 71:29). TLC R_f 0.42 and 0.50 (hexane/EtOAc 5:1); ^1H NMR (400 MHz, $CDCl_3$) δ 1.20−1.80 (m, 18 H), 1.91−2.08 (m, 3 H), 3.39 (m, trans 1 H), 4.51 (dt, *J* = 6.0, 6.0 Hz, 1 H); The ^1H NMR spectrum is in agreement with that of an authentic sample synthesized using I_2 and H_2O_2 according to the literature[4] (*vide infra*).

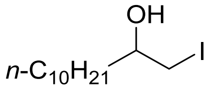

5c-I

1-Iodododecan-2-ol (5c-I). Electrochemical oxidation (2.1 F mol^{-1}) of Bu_4NI (93 mg, 0.25 mmol), subsequent addition of the solution of 1-dodecene (**2c**) (33.9 mg, 0.201 mmol) in CH_2Cl_2 (0.5 mL), and the treatment with NaOH followed by flash chromatography (hexane/EtOAc 10:1) gave the title compound (33.0 mg, 0.106 mmol, 53%). TLC R_f 0.26 (hexane/EtOAc 10:1); ^1H NMR (400 MHz, $CDCl_3$) δ 0.88 (t, *J* = 6.8 Hz, 3 H), 1.10−1.50 (m, 16 H), 1.52−1.58 (m, 2 H), 1.98 (d, *J* = 5.2 Hz, 1 H), 3.23 (dd, *J* = 6.8, 10.0 Hz, 1 H), 3.40 (dd, *J* = 3.6, 10.4 Hz, 1 H), 3.45−3.60 (m, 1 H); ^{13}C NMR (100 MHz, $CDCl_3$) δ 14.1, 16.9, 22.7, 25.6, 29.3, 29.43, 29.47, 29.53, 29.6, 31.9, 36.6, 71.0; HRMS (EI) calcd for $C_{12}H_{25}OBr$ (M^+): 312.0951, found: 312.0958. The ^1H NMR spectrum is in agreement with that in the literature[7].

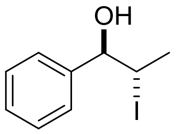

5d-I

(1*R,2*S**)-2-Iodo-1-phenyl-1-propanol** (**5d-I**). Electrochemical oxidation (2.1 F mol^{-1}) of Bu_4NI (87.4 mg, 0.236 mmol), subsequent addition of the solution of (*E*)-β-methylstylene (**(*E*)-2d'**) (21.8 mg, 0.185 mmol) in CH_2Cl_2 (0.5 mL), and the treatment with NaOH followed by flash chromatography (hexane/EtOAc 100:0 to 50:1) gave the title compound (16.9 mg, 0.064 mmol, 35%). TLC R_f 0.35 (hexane/EtOAc 5:1); ^1H NMR (400 MHz, $CDCl_3$) δ 1.74 (d, *J* = 6.8 Hz, 3 H), 2.37(s, 1 H), 4.52 (dq, *J* = 3.6, 7.2 Hz, 1 H), 4.96 (t, *J* = 3.6 Hz, 1 H), 7.29−7.40 (m, 5 H); ^{13}C NMR (100 MHz, $CDCl_3$) δ 21.2, 35.9, 78.5, 126.4, 128.0, 128.4, 139.7; HRMS (EI) calcd for $C_9H_{11}OI$ (M^+): 261.9855, found: 261.9860. Stereochemistry was determined by comparison of the ^1H NMR spectrum with that in the literature[8].

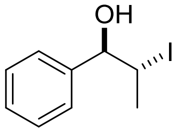

5d'-I

(1*R,2*R**)-2-Iodo-1-phenyl-1-propanol** (**5d'-I**). Electrochemical oxidation (2.1 F mol^{-1}) of Bu_4NI (93.1 mg, 0.252 mmol), subsequent addition of the solution of (*Z*)-β-methylstylene (**(*Z*)-2d**) (22.4 mg, 0.190 mmol) in CH_2Cl_2 (0.5 mL), and the treatment with NaOH followed by flash chromatography (hexane/EtOAc 10:1) gave the title compound (27.0 mg, 0.098 mmol, 51%). TLC R_f 0.13 (hexane/EtOAc 10:1);

^{1}H NMR (400 MHz, $CDCl_3$) δ 1.81 (d, *J* = 6.8 Hz, 3 H), 2.51 (d, *J* = 4.0 Hz, 1 H), 4.39–4.47 (m, 2 H), 7.31–7.41 (m, 5 H); ^{13}C NMR (100 MHz, $CDCl_3$) δ 25.4, 39.1, 79.6, 126.3, 128.4, 128.6, 139.9; HRMS (EI) calcd for $C_9H_{11}OI$ (M^+): 261.9855, found: 261.9856. Stereochemistry was determined by comparison of the ^{1}H NMR spectrum with that in the literature[8].

Synthesis of (5*R,6*R**)-6-iododecan-5-ol (5a-I)**

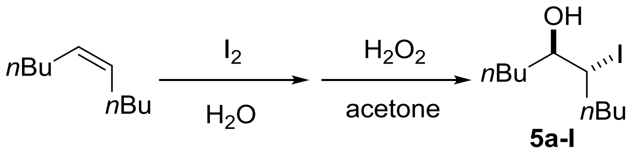

To a round-bottom flask were added iodine (1.07 g, 4.22 mmol), (*Z*)-5-decene (271 mg, 1.94 mmol), H_2O (10 mL) and stirred for 30 min. Then acetone (10 mL), H_2O_2 (31% in H_2O, 400 μL, 3.64 mmol) were added and the mixture was stirred at room temperature for 3 d. Afterwards, the solution was extracted with CH_2Cl_2 (20 mL x 3), dried over Na_2SO_4 and the solvent removed under reduced pressure. The crude product was purified by flash chromatography (hexane/EtOAc 100:0 to 20:1) to obtain (5*R**,6*R**)-6-iododecan-5-ol (**5a**-I) in 91% yield (500 mg, 1.76 mmol). TLC R_f 0.56 (hexane/EtOAc 5:1); ^{1}H NMR (400 MHz, $CDCl_3$) δ 0.92 (t, *J* = 7.2 Hz, 6 H), 1.25–1.46 (m, 6 H), 1.47–1.60 (m, 4 H), 1.62 (d, *J* = 8.4 Hz, 1 H), 1.78–1.88 (m, 1 H), 1.98–2.09 (m, 1 H), 2.85–2.90 (m, 1 H), 4.19 (ddd, *J* = 2.8, 4.8, 9.2 Hz, 1 H); ^{13}C NMR (100 MHz, $CDCl_3$) δ 13.9, 14.0, 22.0, 22.6, 27.7, 32.0, 37.4, 37.7, 50.5, 74.1; HRMS (ESI) calcd for $C_{10}H_{21}OICl$ ($M+Cl^-$): 319.0331, found: 319.0331.

Synthesis of 2-iodocyclododecan-1-ol (5b-I)

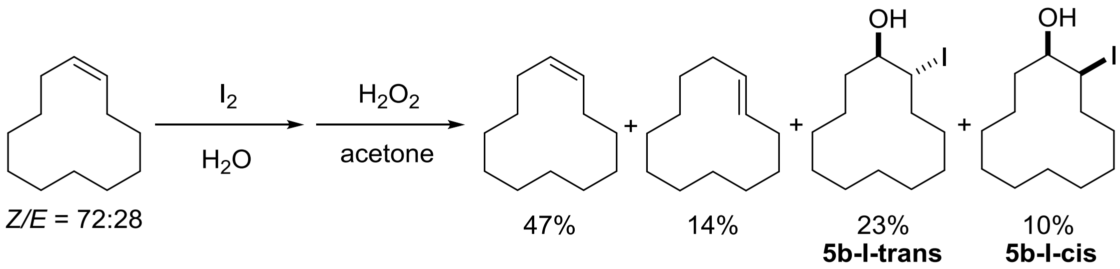

To a round-bottom flask were added iodine (550 mg, 2.16 mmol), cyclododecene (*Z*/*E* = 72:28, 174 mg, 1.05 mmol), and H_2O (5 mL). The mixture was stirred for 30 min at room temperature. After the addition of acetone (5 mL) and H_2O_2 (31% in H_2O, 200 μL, 1.82 mmol), the mixture was stirred at room temperature for 3 d. The solution was extracted with CH_2Cl_2 (20 mL x 3), dried over Na_2SO_4 and the solvent removed under reduced pressur. NMR analysis of the crude product indicated that (*Z*)- and (*E*)-cyclododecenes were recovered in 47%, 14% yield, respectively and that (1*S**,2*S**)-2-iodocyclododecan-1-ol and (1*S**,2*R**)-2-iodocyclododecan-1-ol were obtained in 23%, and 10% yield, respectively. (1*S**,2*S**)-2-Iodocyclododecan-1-ol and (1*S**,2*R**)-2-iodocyclododecan-1-ol were isolated by column chromatography.

(1*R,2*R**)-2-Iodocyclododecan-1-ol (*trans*-5b-I)**: TLC R_f 0.57 (hexane/EtOAc 5:1); ^{1}H NMR (400 MHz,

$CDCl_3$) δ 1.20−1.80 (m, 18 H), 1.88 (d, *J* = 6.4 Hz, 1 H), 1.91−2.01 (m, 1 H), 2.08−2.18 (m, 1 H), 3.39 (m, 1 H), 4.52 (dt, *J* = 6.0, 6.0 Hz, 1 H); ^{13}C NMR (100 MHz, $CDCl_3$) δ 20.9, 23.0, 23.3, 23.4, 23.8, 23.9, 24.0, 24.4, 32.1, 34.9, 47.3, 70.8; HRMS (ESI) calcd for $C_{12}H_{23}IONa^+$ (M+Na$^+$): 333.0686, found: 333.0685.

(1*R,2*S**)-2-iodocyclododecan-1-ol (*cis*-5b-I)**: TLC R_f 0.45 (hexane/EtOAc 5:1); ^{1}H NMR (600 MHz, $CDCl_3$, 50 °C) δ 1.25−1.56 (m, 17 H), 1.71−1.80 (m, 1 H), 1.88 (s, br, 1 H), 2.02 (dt, *J* = 7.2, 7.2 Hz, 2 H), 3.59 (s, br, 1 H), 4.50 (t, *J* = 7.2 Hz, 1 H); ^{13}C NMR (150 MHz, pyridine–d5, 100 °C) δ 22.8, 22.9, 24.91, 24.98, 25.01, 25.4, 25.9, 32.5, 33.8, 45.5, 73.5; HRMS (ESI) calcd for $C_{12}H_{23}OICl^-$ (M+Cl$^-$): 345.0488, found: 345.0490.

4. Synthesis of epoxides

Generation of **1-Br** and **1-I** was conducted in a similar manner to a procedure from ref. [1].

Preparation of 4-pentenyl benzoate

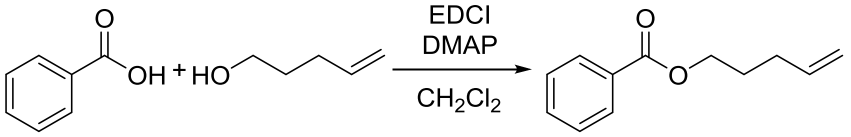

2e

To a round-bottom flask were added 4-pentenol (430 mg, 5.0 mmol), benzoic acid (630 mg, 5.2 mmol), EDCI (1.15 g, 6.0 mmol), DMAP (120 mg, 0.98 mmol), and CH_2Cl_2 (15 mL). The mixture was stirred at room temperature for 2 d. After the addition of water, the solution was extracted with CH_2Cl_2 (20 mL x 3). The solvent was removed under reduced pressure, and the residue was purified through a short column (2 x 4 cm) of silica gel by using hexane/EtOAc (1:1) as an eluent. After removal of the solvent under reduced pressure, the crude product was further purified by flash chromatography (hexane/EtOAc 20:1) to obtain 4-pentenyl benzoate **(2e)** in 96% yield (910 mg, 4.78 mmol). TLC R_f 0.59 (hexane/EtOAc 5:1); ^{1}H NMR (400 MHz, $CDCl_3$) δ 1.88 (tt, *J* = 6.4, 7.6 Hz, 2 H), 2.23 (td, *J* = 6.8, 8.0 Hz, 2 H), 4.34 (t, *J* = 6.4 Hz, 2 H), 5.02 (dd, *J* = 2.0, 10.0 Hz, 1 H), 5.08 (dd, *J* = 2.0, 16.8 Hz, 1 H), 5.86 (tdd, *J* = 6.4, 10.4, 17.2 Hz, 1 H), 7.44 (t, *J* = 7.2 Hz, 2 H), 7.56 (t, *J* = 7.6 Hz, 1 H), 8.05 (d, *J* = 7.6 Hz, 2 H); ^{13}C NMR (100 MHz, $CDCl_3$) δ 27.9, 30.2, 64.3, 115.4, 128.3, 129.5, 130.4, 132.8, 137.5, 166.6; HRMS (EI) calcd for $C_{12}H_{14}O_2Ma$ (M+Na$^+$): 213.0886, found: 213.0883. The ^{1}H NMR spectrum is in agreement with that in the literature[9].

Typical procedure for the generation of 1-Br and the synthesis of epoxides

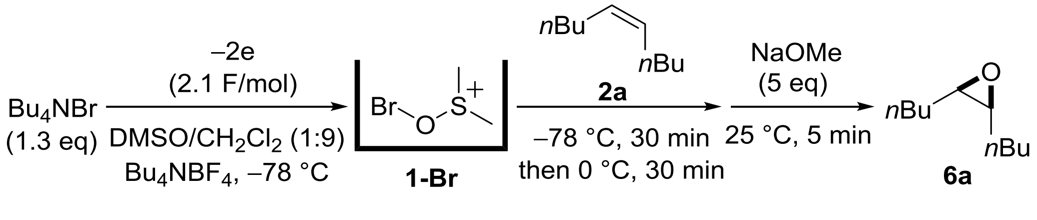

In the anodic chamber were placed Bu_4NBr (80.6 mg, 0.250 mmol), Bu_4NBF_4 (97 mg, 0.29 mmol), DMSO (1 mL), and 0.3 M Bu_4NBF_4/CH_2Cl_2 (9 mL). In the cathodic chamber were placed TfOH (60 µL,

0.68 mmol) and 0.3 M Bu_4NBF_4/CH_2Cl_2 (10 mL). The constant current electrolysis (8.0 mA) was carried out at −78 °C with magnetic stirring until 2.1 F mol^{-1} of electricity was consumed. To the anodic chamber was added a solution of (*Z*)-5-decene (**2a**) (27.7 mg, 0.197 mmol) in CH_2Cl_2 (0.5 mL), and to the cathodic chamber 0.5 mL of CH_2Cl_2 was added at −78 °C. The solution was stirred for 30 min at −78 °C then stirring was continued for 30 min at 0 °C. NaOMe (5.0 M in MeOH, 0.2 mL) was added to both the anodic and the cathodic chambers, and the resulting mixture was warmed to 25 °C and stirred for 5 min. The solution in the anodic chamber was filtered through a short column (2 x 4 cm) of silica gel to remove Bu_4NBF_4 by using Et_2O as an eluent. The GC analysis using hexadecane as an internal standard indicated that (5*R**,6*S**)-5,6-epoxydecane (**6a**) was obtained in 95% yield (0.187 mmol). TLC R_f 0.83 (hexane/EtOAc 5:1); ^1H NMR (400 MHz, $CDCl_3$) δ 0.93 (t, *J* = 7.2 Hz, 6 H), 1.37−1.55 (m, 12 H), 2.88−2.94 (m, 2 H); ^{13}C NMR (100 MHz, $CDCl_3$) δ 14.0, 22.6, 27.5, 28.3, 57.2; HRMS (APCI) calcd for $C_{10}H_{21}O$ $(M+H^+)$: 157.1587, found: 157.1587. Stereochemistry was determined by comparison of the ^1H NMR spectrum with that in the literature[3].

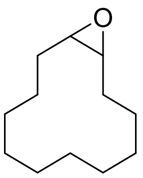

6b

1,2-Epoxycyclododecane (6b). Electrochemical oxidation (2.1 F mol^{-1}) of Bu_4NBr (81.7 mg, 0.254 mmol), subsequent addition of the solution of cyclododecene (**2b**) (*Z*/*E* = 72:28, 32.7 mg, 0.196 mmol) in CH_2Cl_2 (0.5 mL), and treatment with NaOMe followed by flash chromatography (hexane) gave the title compound (22.3 mg, 0.134 mmol, 68%, *cis:trans* = 74:26). TLC R_f 0.54 (hexane/EtOAc 5:1); ^1H NMR (400 MHz, $CDCl_3$) δ 1.20−1.61 (m, 20 H), 1.70−1.87 (m, cis 2 H), 2.15−2.23 (m, trans 2 H), 2.71 (m, trans 2 H), 2.90 (td, *J* = 1.6, 10.0 Hz, cis 2 H). The ^1H NMR spectrum is in agreement with the literature[10].

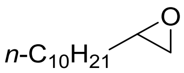

6c

1,2-Epoxydodecane (6c). Electrochemical oxidation (2.1 F mol^{-1}) of Bu_4NBr (80.5 mg, 0.250 mmol), subsequent addition of the solution of 1-dodecene (**2c**) (31.8 mg, 0.189 mmol) in CH_2Cl_2 (0.5 mL), and treatment with NaOMe gave the title compound (0.138 mmol, 73%). The yield was determined by GC analysis using hexadecane as internal standard. TLC R_f 0.63 (hexane/EtOAc 5:1); ^1H NMR (400 MHz, $CDCl_3$) δ 0.88 (t, *J* = 6.8 Hz, 3 H), 1.20−1.38 (m, 14 H), 1.40−1.56 (m, 4 H), 2.46 (dd, *J* = 2.8, 4.8 Hz, 1 H), 2.75 (dd, *J* = 3.6, 4.0 Hz, 1 H), 2.88–2.94 (m, 1 H). The ^1H NMR spectrum is in agreement with the literature[11].

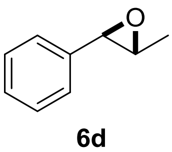

6d

(1*R,2*R**)-1-Phenyl-1,2-epoxypropane (6d).** Electrochemical oxidation (2.1 F mol^{-1}) of Bu_4NBr (82.4 mg, 0.256 mmol), subsequent addition of the solution of (*E*)-β-methylstylene ((***E***)**-2d**) (22.9 mg, 0.194 mmol) in CH_2Cl_2 (0.5 mL), and treatment with NaOMe followed by flash chromatography[12] (hexane) gave

the title compound (13.7 mg, 0.102 mmol, 53%). TLC R_f 0.58 (hexane/EtOAc 5:1); ^{1}H NMR (400 MHz, $CDCl_3$) δ 1.46 (d, *J* = 5.2 Hz, 3 H), 3.04 (dq, *J* = 2.0, 5.2 Hz, 1 H), 3.58 (d, *J* = 1.6 Hz, 2 H), 7.24−7.39 (m, 5 H). The ^{1}H NMR spectrum is in agreement with the literature[13].

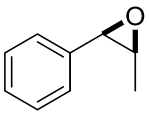

6d'

(1*R,2*S**)-1-Phenyl-1,2-epoxypropane (6d').** Electrochemical oxidation (2.1 F mol^{-1}) of Bu_4NBr (81.7 mg, 0.254 mmol), subsequent addition of the solution of (*Z*)-β-methylstylene (**(*Z*)-2d**) (22.9 mg, 0.194 mmol) in CH_2Cl_2 (0.5 mL), and treatment with NaOMe followed by flash chromatography[12] (hexane) gave the title compound (15.7 mg, 0.117 mmol, 60%). TLC R_f 0.56 (hexane/EtOAc 5:1); ^{1}H NMR (400 MHz, $CDCl_3$) δ 1.09 (d, *J* = 5.6 Hz, 3 H), 3.34 (dq, *J* = 4.4, 5.2 Hz, 1 H), 4.07 (d, *J* = 4.4 Hz, 1 H) 7.26−7.40 (m, 5 H). The ^{1}H NMR spectrum is in agreement with the literature[13].

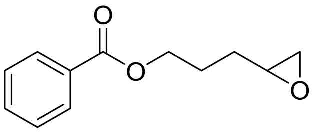

6e

4,5-Epoxypentenyl benzoate (6e). Electrochemical oxidation (2.1 F mol^{-1}) of Bu_4NBr (81.6 mg, 0.253 mmol), subsequent addition of the solution of 4-pentenyl benzoate (**2f**) (37.2 mg, 0.196 mmol) in CH_2Cl_2 (0.5 mL), and treatment with NaOMe followed by flash chromatography[12] (hexane/EtOAc 100:0 to 20:1) gave the title compound (21.0 mg, 0.102 mmol, 52%). TLC R_f 0.29 (hexane/EtOAc 5:1); ^{1}H NMR (400 MHz, $CDCl_3$) δ 1.62−1.82 (m, 2 H), 1.88−2.04 (m, 2 H), 2.52 (dd, *J* = 2.8, 5.2 Hz, 1 H), 2.78 (dd, *J* = 4.0, 5.2 Hz, 1 H), 2.98−3.02 (m, 1 H), 4.38 (dt, *J* = 2.4, 6.4 Hz, 2 H), 7.44 (t, *J* = 8.0 Hz, 2 H), 7.56 (t, *J* = 7.6 Hz, 1 H), 8.04 (d, *J* = 8.4 Hz, 2 H); ^{13}C NMR (100 MHz, $CDCl_3$) δ 25.3, 29.1, 47.0, 51.7, 64.4, 128.3, 129.5, 130.2, 132.9, 166.5; HRMS (ESI) calcd for $C_7H_7O_2$ (M−H^+): 207.1016, found: 207.1012. The ^{1}H NMR spectrum is in agreement with the literature[14].

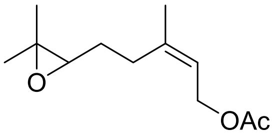

6f

6,7-Epoxyneryl acetate (6f). Electrochemical oxidation (2.1 F mol^{-1}) of Bu_4NBr (80.7 mg, 0.251 mmol), subsequent addition of the solution of neryl acetate (**2f**) (357.3 mg, 0.190 mmol) in CH_2Cl_2 (0.5 mL), and treatment with NaOMe followed by flash chromatography[12] (hexane/EtOAc 100:0 to 50:1) gave the title compound (19.7 mg, 0.093 mmol, 49%). TLC R_f 0.30 (hexane/EtOAc 5:1); ^{1}H NMR (400 MHz, $CDCl_3$) δ 1.27 (s, 3 H), 1.31 (s, 3 H), 1.56−1.71 (m, 2 H), 1.79 (s, 3 H), 2.05 (s, 3 H) 2.23−2.29 (m, 2 H), 2.71 (t, *J* = 6.0 Hz, 1 H), 4.59 (d, *J* = 7.6 Hz, 2 H), 5.41 (t, *J* = 7.2 Hz, 1 H); ^{13}C NMR (100 MHz, $CDCl_3$) δ 18.7, 21.0, 23.4, 24.8, 27.5, 28.8, 58.4, 60.8, 63.7, 119.7, 141.7, 171.0; HRMS (ESI) calcd for $C_{12}H_{20}O_3Na$ (M+Na^+): 235.1305, found: 235,1299. The ^{1}H NMR spectrum is in agreement with the literature[15].

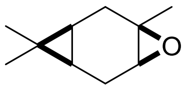

6g

(1*S*,3*R*,4*S*,6*R*)-3,4-Epoxycarane (6g). Electrochemical oxidation (2.1 F mol^{-1}) of Bu_4NBr (80.4 mg, 0.250 mmol), subsequent addition of the solution of (+)-3-carene (**2g**) (26.1 mg, 0.192 mmol) in CH_2Cl_2 (0.5 mL), and treatment with NaOMe followed by flash chromatography[12] (hexane) gave the title compound. (20.1 mg, 0.132 mmol, 69%). TLC R_f 0.50 (hexane/EtOAc 5:1); ^{1}H NMR (400 MHz, $CDCl_3$) δ 0.52 (dt, *J* = 2.0, 9.2 Hz, 1 H), 0.57 (dt, *J* = 2.0, 9.2 Hz, 1 H), 0.92 (s, 3 H), 0.96 (s, 3 H), 1.30 (s, 3 H), 1.78 (d, *J* = 15.6 Hz, 2 H), 2.06 (dd, *J* = 9.2, 16.4 Hz, 1 H) 2.28 (ddd, *J* = 5.6, 9.2, 16.4 Hz, 1 H) 2.88 (d, 5.6 Hz, 1 H); ^{13}C NMR (100 MHz, $CDCl_3$) δ 14.7, 17.3, 17.5, 18.3, 19.4, 23.9, 24.7, 29.1, 55.9, 58.2; HRMS (ESI) calcd for $C_{10}H_{17}O$ $(M+H^+)$: 153.1274, found: 153.1270. Stereochemistry was determined by comparison of the ^{1}H NMR spectrum with that in the literature[16].

Typical procedure for the generation of 1-I and the synthesis of epoxides

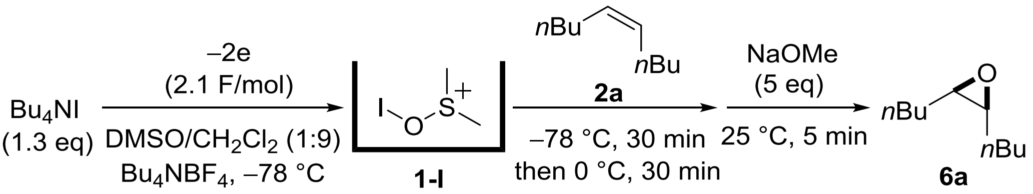

In the anodic chamber were placed Bu_4NI (91.7 mg, 0.248 mmol), Bu_4NBF_4 (102 mg, 0.3 mmol), DMSO (1 mL), and 0.3 M Bu_4NBF_4/CH_2Cl_2 (9 mL). In the cathodic chamber were placed TfOH (60 μL, 0.68 mmol) and 0.3 M Bu_4NBF_4/CH_2Cl_2 (10 mL). The constant current electrolysis (8.0 mA) was carried out at −78 °C with magnetic stirring until 2.1 F mol^{-1} of electricity was consumed. To the anodic chamber was added a solution of (*Z*)-5-decene (**2a**) (27.9 mg, 0.199 mmol) in CH_2Cl_2 (0.5 mL), and to the cathodic chamber 0.5 mL of CH_2Cl_2 was added at −78 °C. The solution was stirred for 30 min at −78 °C then stirring was continued for 30 min at 0 °C. NaOMe (5.0 M in MeOH, 0.2 mL) was added to both the anodic and the cathodic chambers, and the resulting mixture was warmed to 25 °C and stirred for 5 min. The solution in the anodic chamber was filtered through a short column (2 x 4 cm) of silica gel to remove Bu_4NBF_4 by using Et_2O as an eluent. The GC analysis using hexadecane as an internal standard indicated that (5*R**,6*S**)-5,6-epoxydecane (**6a**) was obtained in 96% yield (0.191 mmol). TLC R_f 0.83 (hexane/EtOAc 5:1); ^{1}H NMR (400 MHz, $CDCl_3$) δ 0.93 (t, *J* = 7.2 Hz, 6 H), 1.37−1.55 (m, 12 H), 2.88−2.94 (m, 2 H). ^{13}C NMR (100 MHz, $CDCl_3$) δ14.0, 22.6, 27.5, 28.3, 57.2; HRMS (APCI) calcd for $C_{10}H_{21}O$ $(M+H^+)$: 157.1587, found: 157.1587. Stereochemistry was determined by comparison of the ^{1}H NMR spectrum with that in the literature[3].

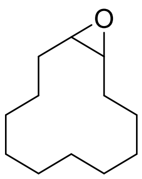

6b

1,2-Epoxycyclododecane (6b). Electrochemical oxidation (2.1 F mol^{-1}) of Bu_4NI (92.5 mg, 0.250 mmol), subsequent addition of the solution of cyclododecene (**2b**) (*Z*/*E* = 72:28, 33.3 mg, 0.200 mmol) in CH_2Cl_2 (0.5 mL), and treatment with NaOMe followed by flash chromatography (hexane/EtOAc 50:1) gave the title compound (32.6 mg, 0.179 mmol, 89%, *cis:trans* = 74:26). TLC R_f 0.54 (hexane/EtOAc 5:1); ^{1}H NMR (400 MHz, $CDCl_3$) δ 1.20−1.61 (m, 20 H), 1.70−1.87 (m, cis 2 H), 2.15−2.23 (m, trans 2 H), 2.71 (m, trans 2 H), 2.90 (td, *J* = 1.6, 10.0 Hz, cis 2 H). The ^{1}H NMR spectrum is in agreement with the literature[10].

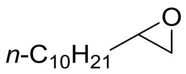

6c

1,2-Epoxydodecane (6c). Electrochemical oxidation (2.1 F mol^{-1}) of Bu_4NI (91.5 mg, 0.248 mmol), subsequent addition of the solution of 1-dodecene (**2c**) (31.8 mg, 0.189 mmol) in CH_2Cl_2 (0.5 mL), and treatment with NaOMe gave the title compound(0.162 mmol, 86%, The yield was determined by GC analysis using hexadecane as internal standard. TLC R_f 0.63 (hexane/EtOAc 5:1); ^{1}H NMR (400 MHz, $CDCl_3$) δ 0.88 (t, *J* = 6.8 Hz, 3 H), 1.20−1.38 (m, 14 H), 1.40−1.56 (m, 4 H), 2.46 (dd, *J* = 2.8, 4.8 Hz, 1 H), 2.75 (dd, *J* = 3.6, 4.0 Hz, 1 H), 2.88−2.94 (m, 1 H). The ^{1}H NMR spectrum is in agreement with the literature[11].

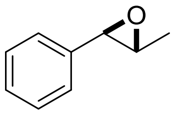

6d

(1*R,2*R**)-1-Phenyl-1,2-epoxypropane (6d).** Electrochemical oxidation (2.1 F mol^{-1}) of Bu_4NI (148 mg, 0.401 mmol), subsequent addition of the solution of (*E*)-β-methylstylene (**(*E*)-2d**) (22.8 mg, 0.193 mmol) in CH_2Cl_2 (0.5 mL), and treatment with NaOMe followed by flash chromatography[12] (hexane) gave the title compound (10.0 mg, 0.074 mmol, 38%). TLC R_f 0.58 (hexane/EtOAc 5:1); ^{1}H NMR (400 MHz, $CDCl_3$) δ 1.46 (d, *J* = 5.2 Hz, 3 H), 3.04 (dq, *J* = 2.0, 5.2 Hz, 1 H), 3.58 (d, *J* = 1.6 Hz, 2 H), 7.24−7.39 (m, 5 H); The ^{1}H NMR spectrum is in agreement with the literature[13].

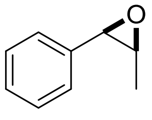

6d'

(1*R,2*S**)-1-Phenyl-1,2-epoxypropane (6d').** Electrochemical oxidation (2.1 F mol^{-1}) of Bu_4NI (149 mg, 0.403 mmol), subsequent addition of the solution of (*Z*)-β-methylstylene (**(*Z*)-2d**) (23.6mg, 0.200 mmol) in CH_2Cl_2 (0.5 mL), and treatment with NaOMe followed by flash chromatography[9] (hexane) gave the title compound (134 mg, 0.134 mmol, 67%). TLC R_f 0.56 (hexane/EtOAc 5:1); ^{1}H NMR (400 MHz, $CDCl_3$) δ 1.09 (d, *J* = 5.6 Hz, 3 H), 3.34 (dq, *J* = 4.4, 5.2 Hz, 1 H), 4.07 (d, *J* = 4.4 Hz, 1 H) 7.26−7.40 (m, 5 H). The ^{1}H NMR spectrum is in agreement with the literature[13].

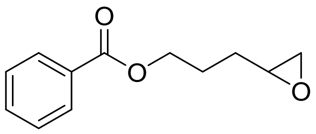

6e

4,5-Epoxypentenyl benzoate (6e). Electrochemical oxidation (2.1 F mol^{-1}) of Bu_4NI (91.0 mg, 0.246 mmol), subsequent addition of the solution of 4-penteneyl benzoate (**2f**) (39.0 mg, 0.205 mmol) in CH_2Cl_2 (0.5 mL), and treatment with NaOMe followed by flash chromatography[12] (hexane/EtOAc 100:0 to 20:1) gave the title compound (24.0 mg, 0.116 mmol, 57%). TLC R_f 0.29 (hexane/EtOAc 5:1); ^{1}H NMR (400 MHz, $CDCl_3$) δ 1.62−1.82 (m, 2 H), 1.88−2.04 (m, 2 H), 2.52 (dd, *J* = 2.8, 5.2 Hz, 1 H), 2.78 (dd, *J* = 4.0, 5.2 Hz, 1 H), 2.98−3.02 (m, 1 H), 4.38 (dt, *J* = 2.4, 6.4 Hz, 2 H), 7.44 (t, *J* = 8.0 Hz, 2 H), 7.56 (t, *J* = 7.6 Hz, 1 H), 8.04 (d, *J* = 8.4 Hz, 2 H); ^{13}C NMR (100 MHz, $CDCl_3$) δ 25.3, 29.1, 47.0, 51.7, 64.4, 128.3, 129.5, 130.2, 132.9, 166.5; HRMS (ESI) calcd for $C_7H_7O_2$ (M+H$^+$): 207.1016, found: 207.1012. The ^{1}H NMR spectrum is in agreement with the literature[14].

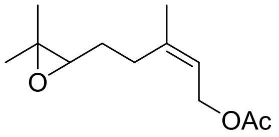

6f

6,7-Epoxyneryl acetate (6f). Electrochemical oxidation (2.1 F mol^{-1}) of Bu_4NI (92.8 mg, 0.251 mmol), subsequent addition of the solution of neryl acetate (**2f**) (35.6 mg, 0.182 mmol) in CH_2Cl_2 (0.5 mL), and treatment with NaOMe followed by flash chromatography[12] (hexane/EtOAc 100:0 to 20:1) gave the title compound (18.2 mg, 0.086 mmol, 47%). TLC R_f 0.30 (hexane/EtOAc 5:1); ^{1}H NMR (400 MHz, $CDCl_3$) δ 1.27 (s, 3 H), 1.31 (s, 3 H), 1.56−1.71 (m, 2 H), 1.79 (s, 3 H), 2.05 (s, 3 H) 2.23−2.29 (m, 2 H), 2.71 (t, *J* = 6.0 Hz, 1 H), 4.59 (d, *J* = 7.6 Hz, 2 H), 5.41 (t, *J* = 7.2 Hz, 1 H); ^{13}C NMR (100 MHz, $CDCl_3$) δ 18.7, 21.0, 23.4, 24.8, 27.5, 28.8, 58.4, 60.8, 63.7, 119.7, 141.7, 171.0; HRMS (ESI) calcd for $C_{12}H_{20}O_3Na$ (M+Na$^+$): 235.1305, found: 235,1299. The ^{1}H NMR spectrum is in agreement with the literature[15].

5. Synthesis of ^{18}O-labelled epoxide

Synthesis of ^{18}O-labelled DMSO[17]

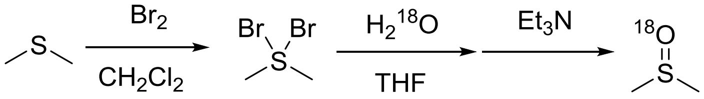

To a round-bottom flask were added dimethylsulfide (2.6 mL, 33 mmol) and CH_2Cl_2 (10 mL). Bromine (1.8 mL, 33 mmol) was added dropwise to the solution at 0 °C over a period of 30 min. The mixture was stirred at 0 °C for 30 min to precipitate a yellow solid material. The liquid phase was removed by syringe and the solid material washed with dry CH_2Cl_2 under an Ar atmosphere. THF (10 mL) was added, followed by the dropwise addition of $H_2{}^{18}O$ (242 mg, 12.1 mmol) at 0 °C over a period of 5 min. After stirring at 0 °C for 1 h, Et_3N (6.4 mL) was added to the mixture. The precipitate of triethylamine hydrobromide was separated by filtration and was washed twice with CH_2Cl_2. The combined yellow filtrate and washings were evaporated under reduced pressure to remove the solvents. The residue was purified by flash chromatography (hexane/EtOAc 1:1 to 0:100 to EtOAc/MeOH 10:1). After Kugelrohr distillation (20 mmHg, 80 °C), ^{18}O-labelled DMSO was obtained in 34% yield (332 mg, 4.14 mmol). TLC R_f 0.05 (EtOAc); ^{1}H NMR (400 MHz, $CDCl_3$) δ 2.62 (s, 6 H); ^{13}C NMR (100 MHz, $CDCl_3$) δ 40.5; HRMS (ESI) calcd for $C_4H_{12}{}^{18}O_2S$ (2M+H$^+$): 161.0436, found: 161.0433.

Synthesis of ^{18}O-labelled epoxide 6c

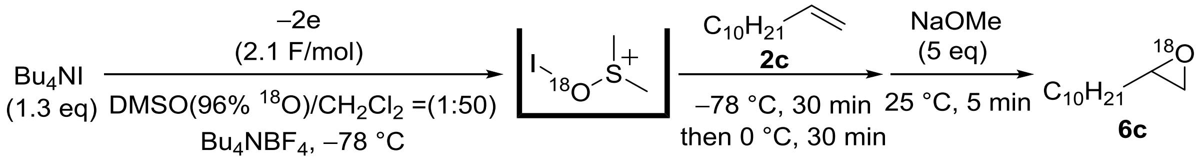

In the anodic chamber were placed Bu_4NI (91.8 mg, 0.248 mmol), DMSO (96% ^{18}O, 0.2 mL), and 0.3 M Bu_4NBF_4/CH_2Cl_2 (10 mL). In the cathodic chamber were placed TfOH (60 μL, 0.68 mmol) and 0.3 M Bu_4NBF_4/CH_2Cl_2 (10 mL). The constant current electrolysis (8.0 mA) was carried out at −78 °C with magnetic stirring until 2.1 F mol^{-1} of electricity was consumed. To the anodic chamber was added a solution of 1-dodecene (**2c**) (32.5 mg, 0.193 mmol) in CH_2Cl_2 (0.5 mL), and to the cathodic chamber was added 0.5 mL of CH_2Cl_2 at −78 °C. The solution was stirred for 30 min at −78 °C then stirring was continued for 30 min at 0 °C. NaOMe (5.0 M in MeOH, 0.2 mL) was added to both the anodic and cathodic chambers, and the resulting mixture was warmed to 25 °C and stirred for 5 min. The solution in the anodic chamber was filtered through a short column (2 x 4 cm) of silica gel to remove Bu_4NBF_4 by using Et_2O as an eluent. After removal of the solvent under reduced pressure the crude product was purified by flash chromatography[9] (hexane) to obtain ^{18}O-labelled 1,2-epoxydodecane (**6c**) in 81% yield (29.0 mg, 0.155 mmol). TLC R_f 0.67 (hexane/EtOAc 5:1); ^{1}H NMR (400 MHz, $CDCl_3$) δ 0.88 (t, *J* = 6.8 Hz, 6 H), 1.22–1.56 (m, 18 H), 2.46 (dd, *J* = 2.8, 5.2 Hz, 1 H), 2.74 (dd, *J* = 4.0, 5.2 Hz, 1 H), 2.88–2.93 (m, 1 H); ^{13}C NMR (100 MHz, $CDCl_3$) δ 14.1, 22.7, 26.0, 29.3, 29.4, 29.54, 29.57, 31.9, 32.5, 47.1, 52.4; HRMS (APCI) calcd for $C_{12}H_{25}{}^{18}O$ (M+H$^+$): 187.1942, found: 187.1940.

6. References

(1) Ashikari, Y.; Shimizu, A.; Nokami, T.; Yoshida, J. *J. Am. Chem. Soc.* **2013**, *135*, 16070.

(2) Van Ende, D.; Krief, A. *Tetrahedron Lett.* **1975**, *31*, 2709.

(3) Brimeyer, M.; Mehrota, A.; Quici, S.; Nigam, A.; Regen, S. L. *J. Org. Chem.* **1980**, *45*, 4255.

(4) Sumrell, G.; Wyman, B. M.; Howell, R. G.; Harvey, M. C. *Can. J. Chem.* **1964**, *42*, 2710.

(5) Haufe, G.; Muhlstadt, M. *Monatsh. Chem.* **1976**, *107*, 653.

(6) Macharla, A. K.; Nappunni, R. C.; Nama, N. *Tetrahedron Lett.* **2012**, *53*, 1401.

(7) Kuroboshi, M.; Hiyama, T. *Bull. Chem. Soc. Jpn.* **1995**, *68*, 1799.

(8) Schmid, G. H.; Gordon, J. W. *J. Org. Chem.* **1983**, *48*, 4010.

(9) Wang, X.; Ye, Y.; Zhang, S.; Feng, J.; Xu, Y.; Zhang, Y.; Wang, J. *J. Am. Chem. Soc.* **2011**, *133*, 16410.

(10) Terent'ev, A. O.; Boyarinova, K. A.; Nikishin, G. I. *Russ. J. Gen. Chem.* **2008**, *78*, 592.

(11) McPake, C. B.; Murray, C. B.; Sandford, G. *Tetrahedron Lett*. **2009**, *50*, 1674.

(12) Flash chromatography was carried out on a silica gel (Fuji Silysia Chemical Ltd., CHROMATOREX FL100DX, spherical, pH = 8.7, 100 μm)

(13) Koya, S.; Nishioka, Y.; Mizoguchi, H.; Uchiba, T.; Katsuki, T. *Angew. Chem. Int. Ed.* **2012**, *51*, 8243.

(14) Wu, Y.; Shen, X.; Yang, Y.; Hu, Q.; Huang, J. *J. Org. Chem.* **2004**, *69*, 3857.

(15) Fillion, E.; Beingessner, R. L. *J. Org. Chem.* **2003**, *68*, 9485.

(16) Cimarelli, C.; Fratoni, D.; Palmieri, G. *Tetrahedron Asymmetry* **2011**, *22*, 603.

(17) Fenselau, A. H.; Moffatt, J. G. *J. Am. Chem. Soc.* **1966**, *88*, 1762.

7. NMR spectra

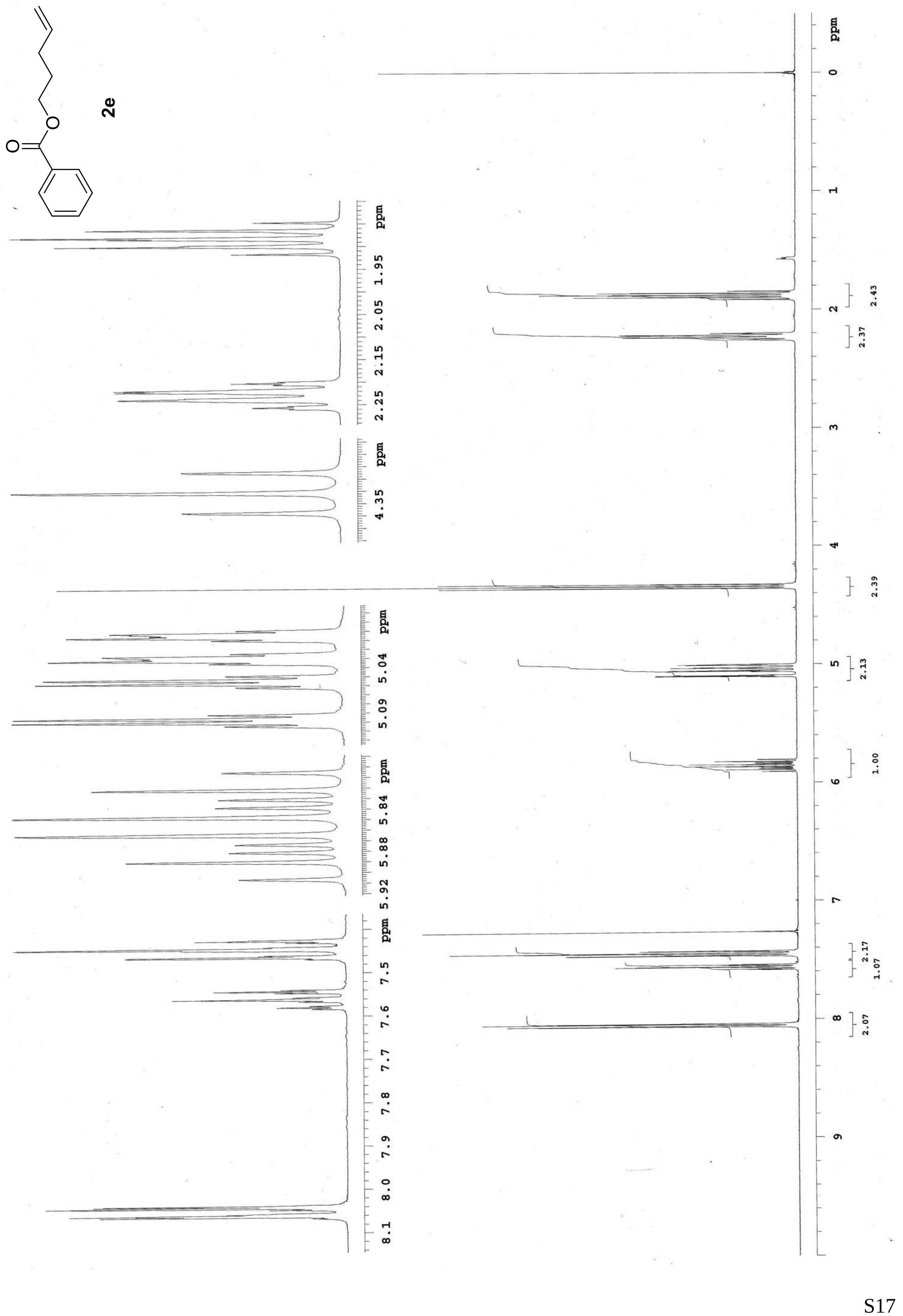

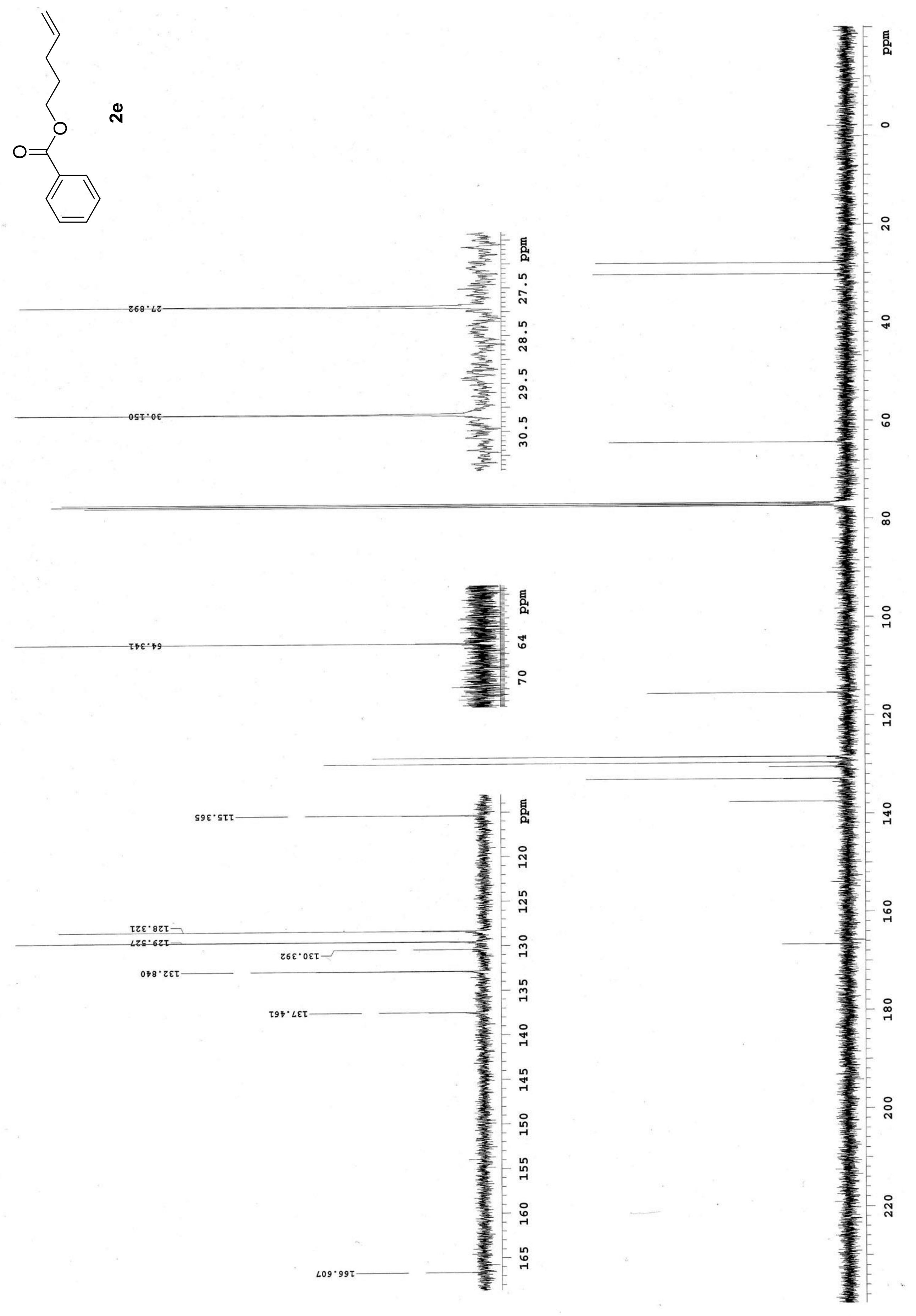
2e
27.892
30.150
64.341
115.365
128.321
129.527
130.392
132.840
137.461
166.607
ppm

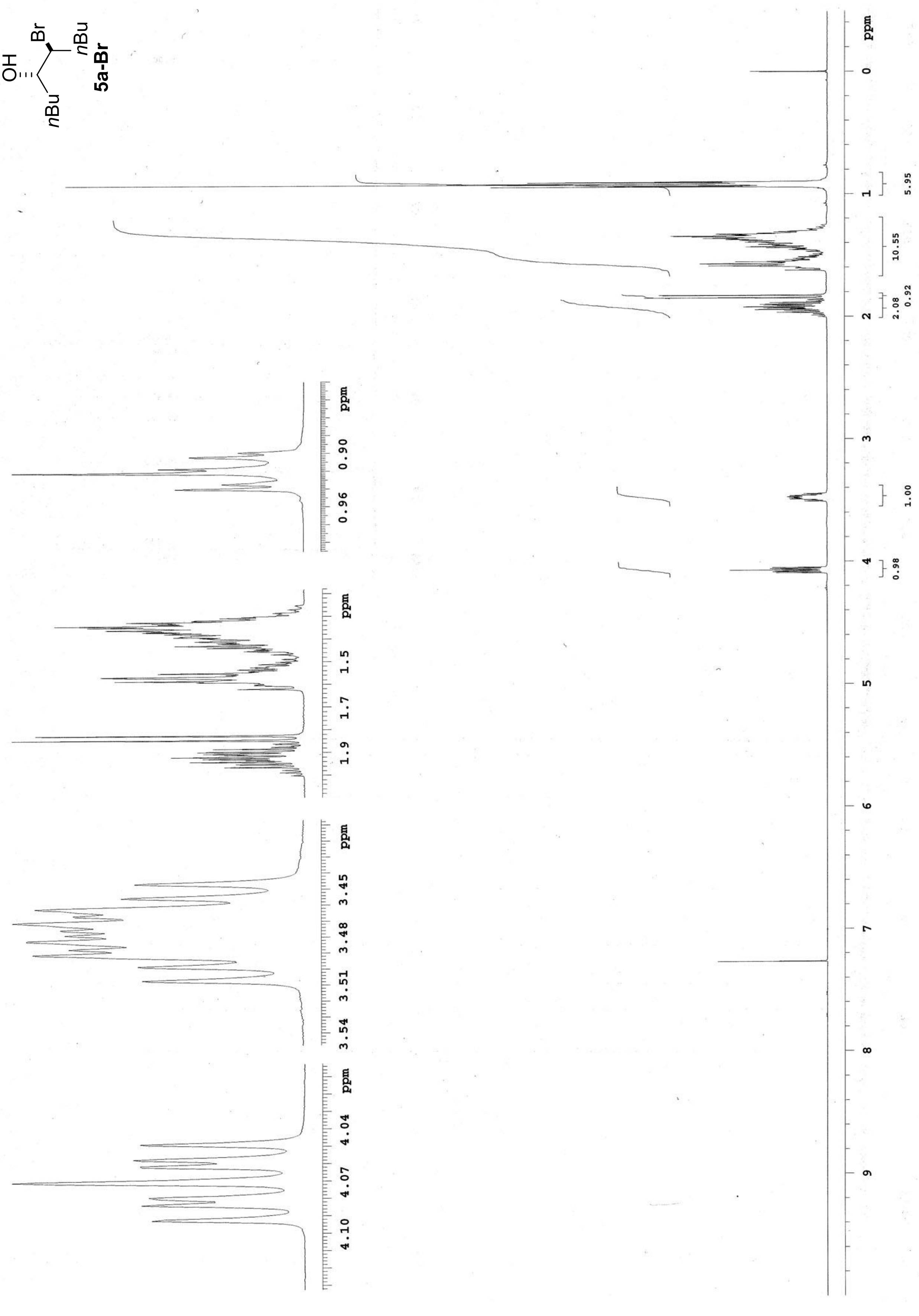

5a-Br
ppm
5.95
10.55
2.08
0.92
1.00
0.98

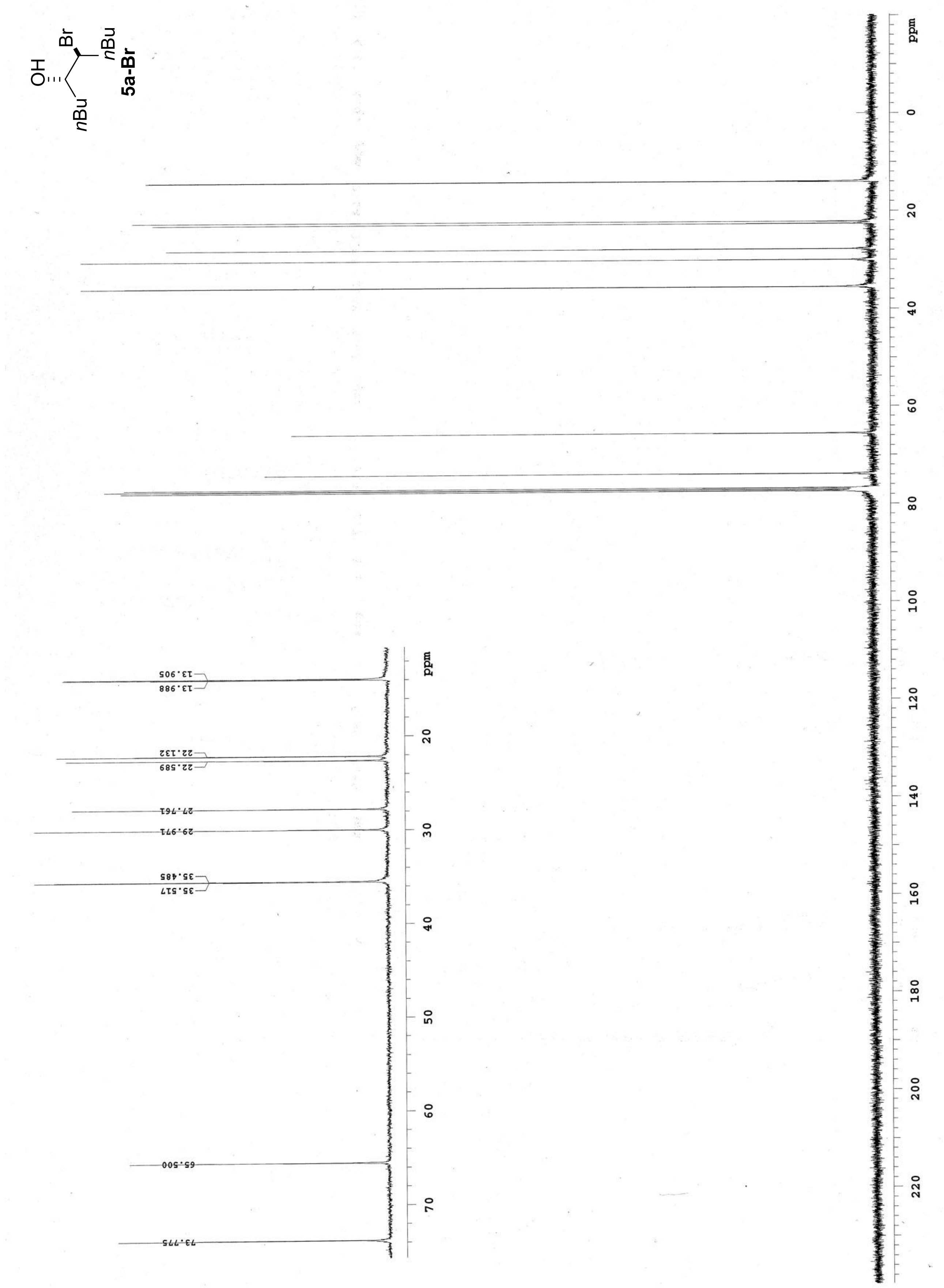
OH
Br
nBu
nBu
5a-Br
13.905
13.988
22.132
22.589
27.761
29.971
35.485
35.517
65.500
73.775
ppm

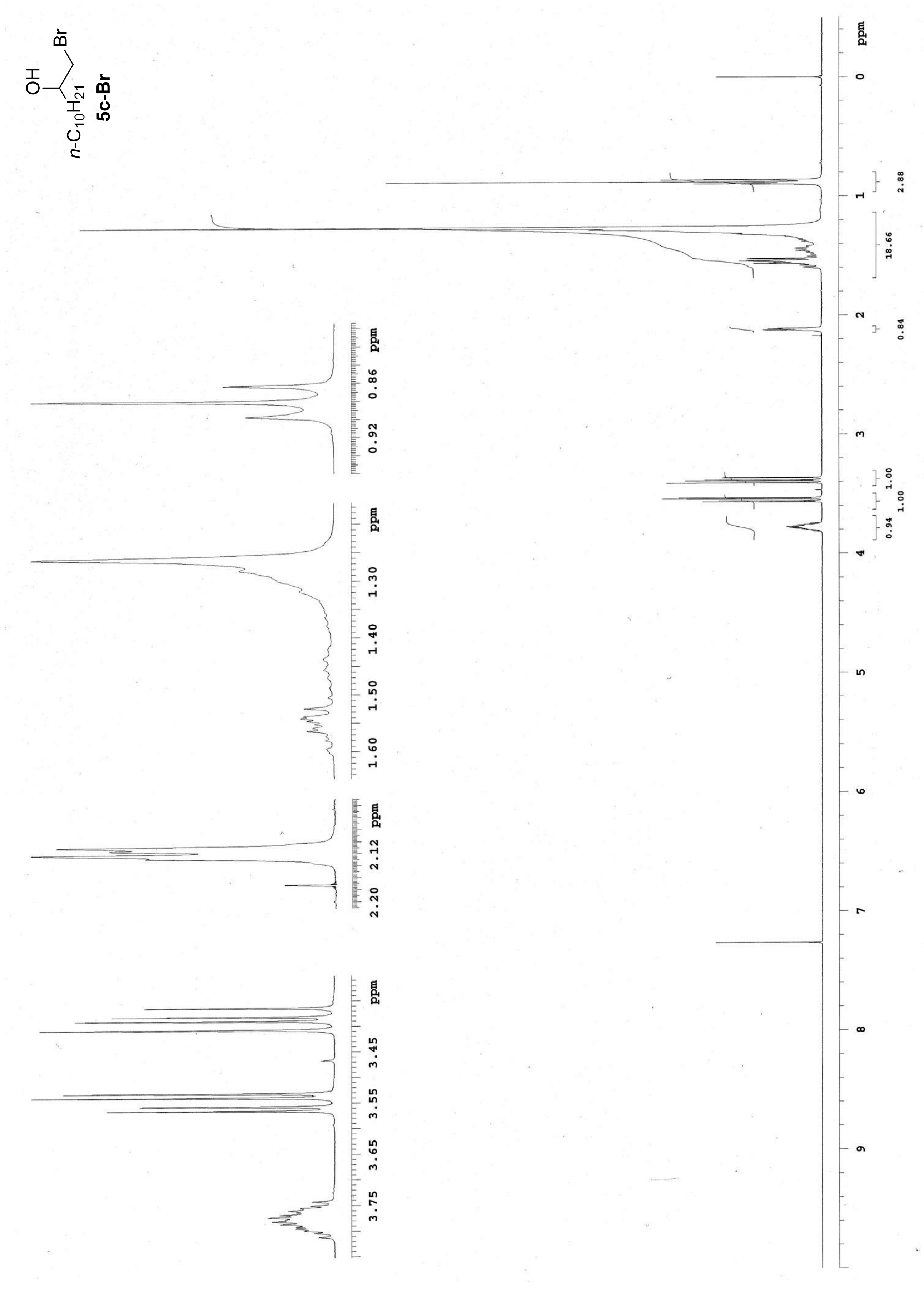
OH
Br
n-C10H21
5c-Br
ppm
2.88
18.66
0.84
1.00
1.00
0.94
0.86
0.92
1.30
1.40
1.50
1.60
2.12
2.20
3.45
3.55
3.65
3.75

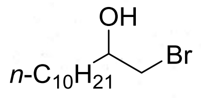
OH
Br
n-C10H21

5c-Br

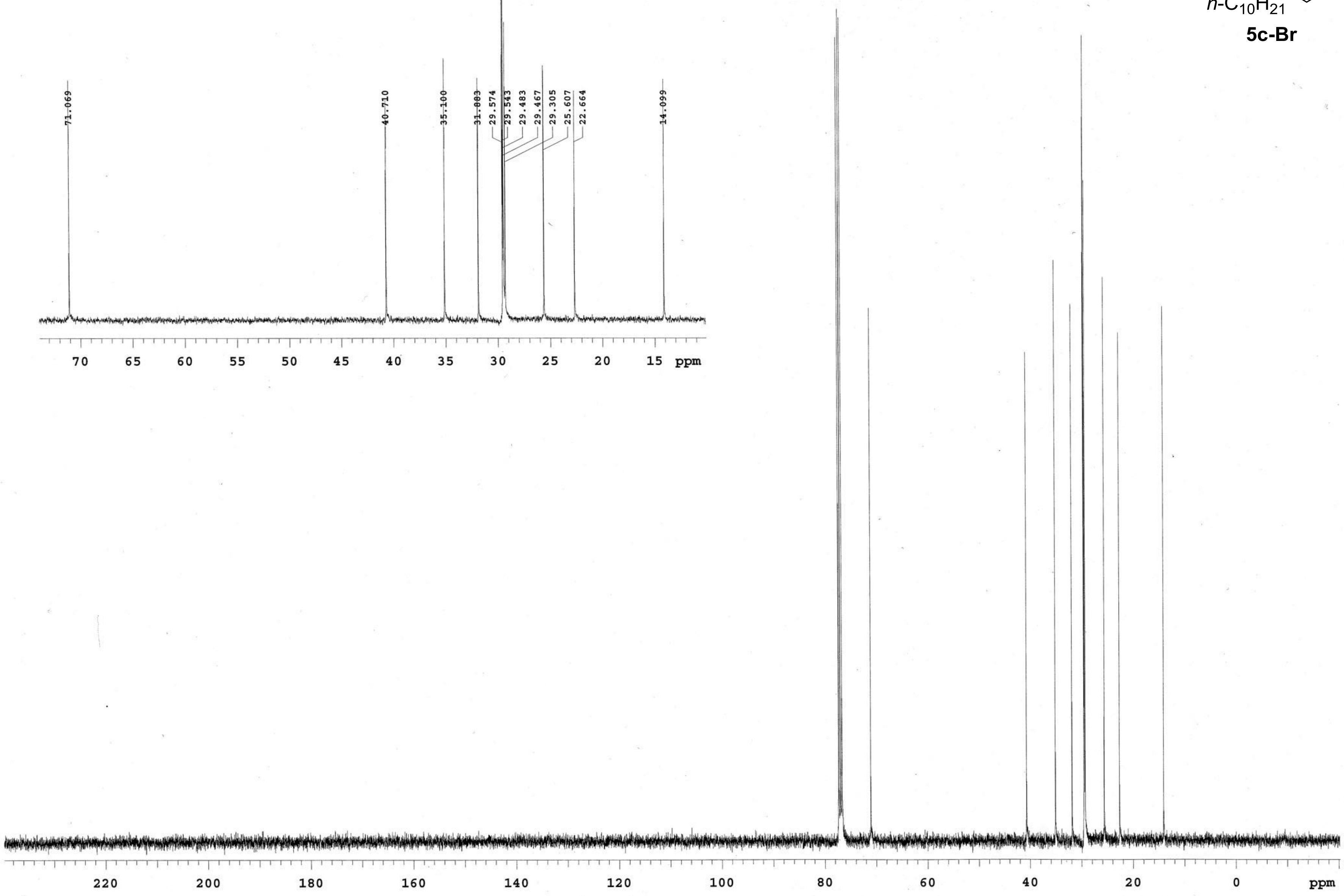
71.069
40.710
35.100
31.883
29.574
29.543
29.483
29.467
29.305
25.607
22.664
14.099
70
65
60
55
50
45
40
35
30
25
20
15
ppm
220
200
180
160
140
120
100
80
60
40
20
0
ppm

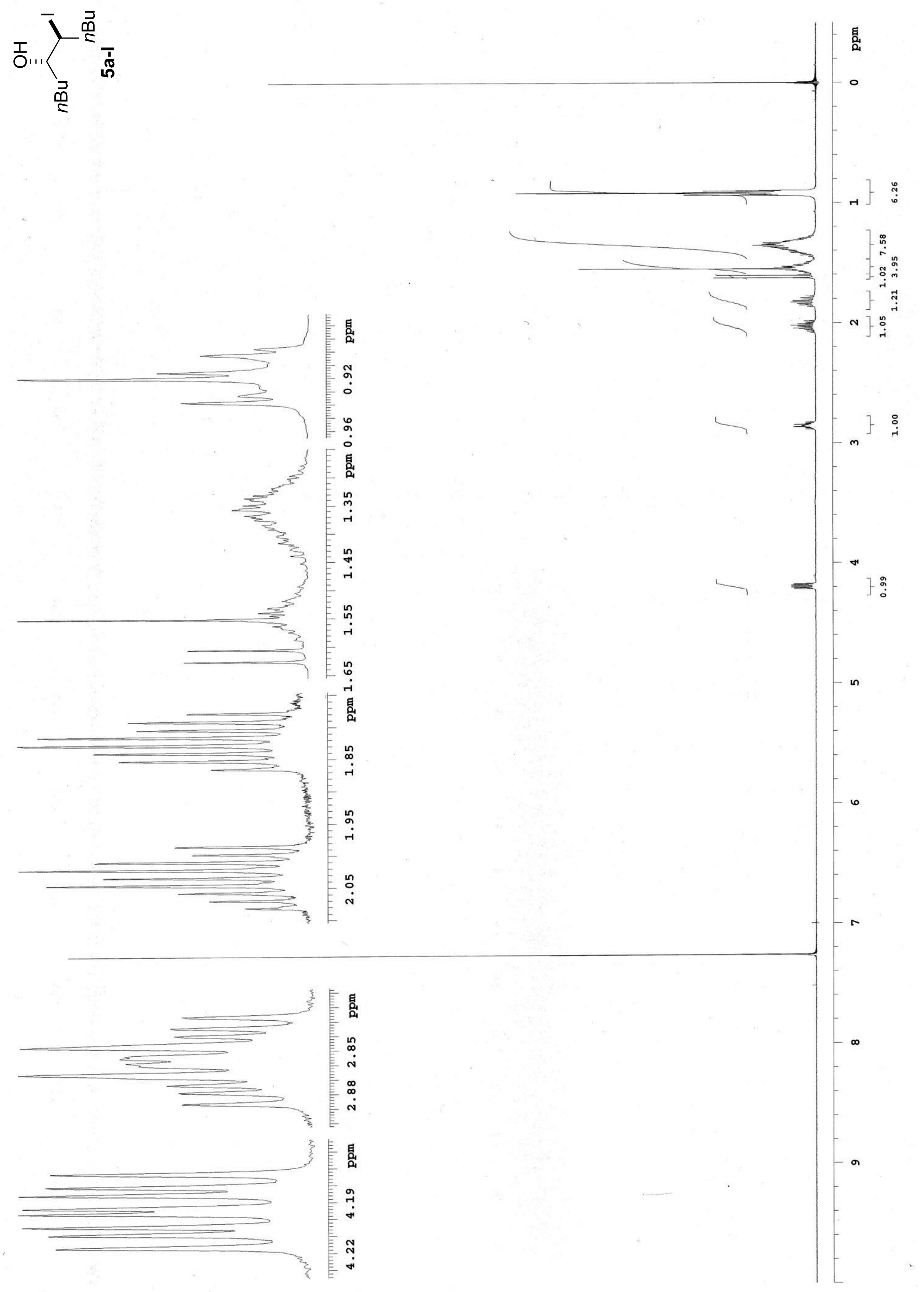

5a-I

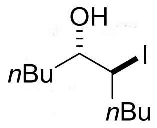

5a-I

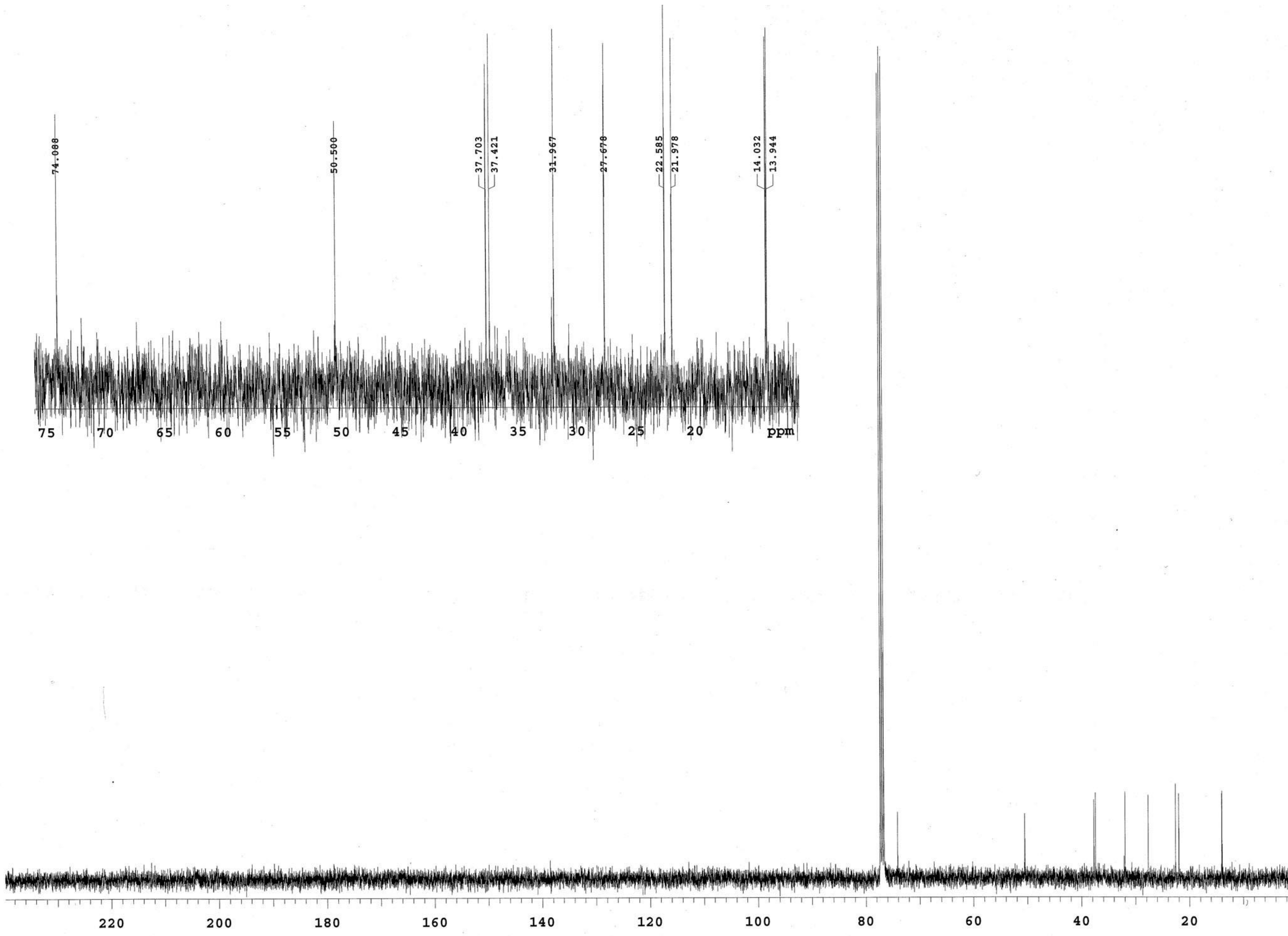
74.088
50.500
37.703
37.421
31.967
27.678
22.585
21.978
14.032
13.944
75
70
65
60
55
50
45
40
35
30
25
20
ppm
220
200
180
160
140
120
100
80
60
40
20
0
ppm

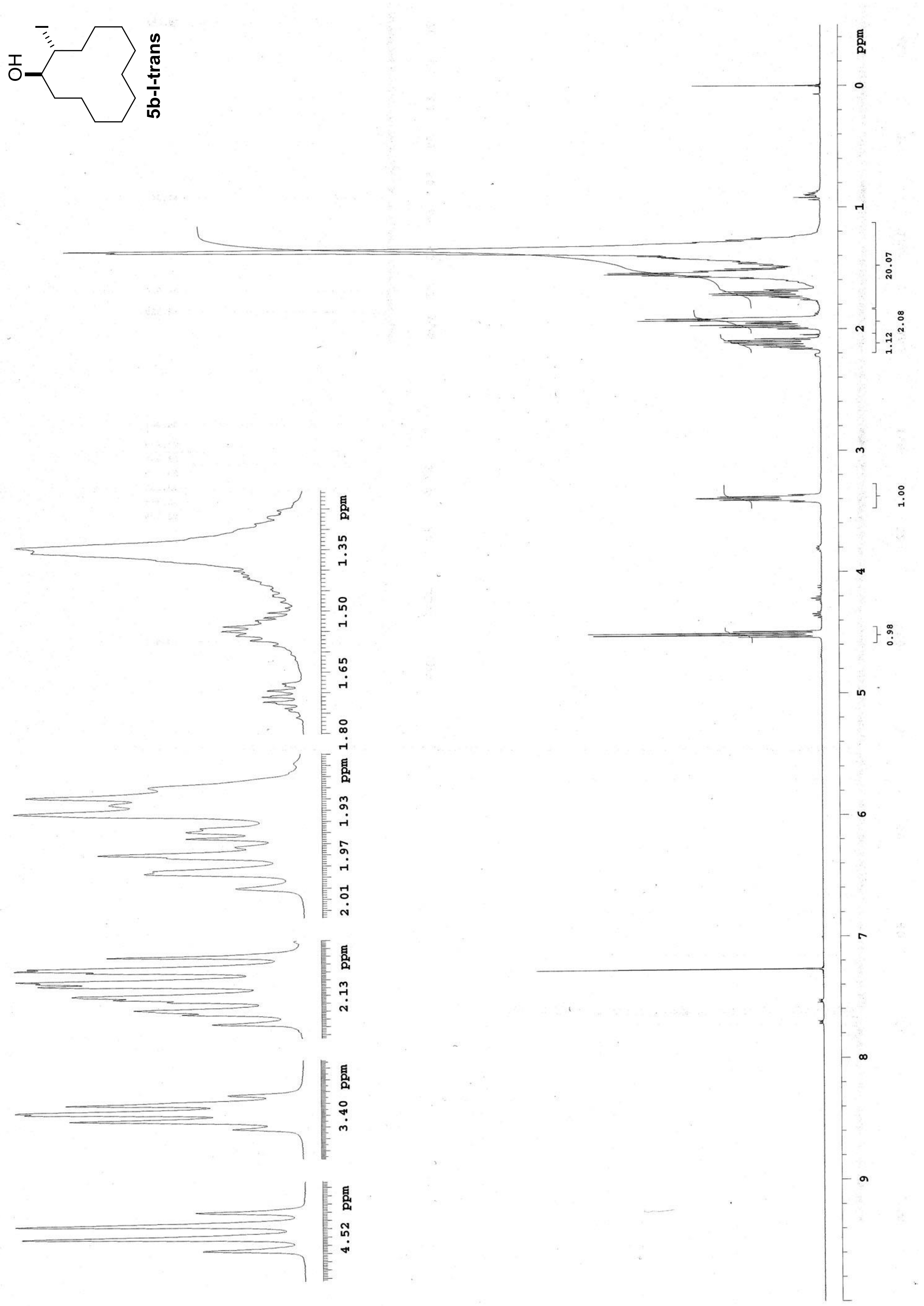
5b-I-trans
OH
I
ppm
20.07
1.12
2.08
1.00
0.98
1.35
1.50
1.65
1.80
1.93
1.97
2.01
2.13
3.40
4.52

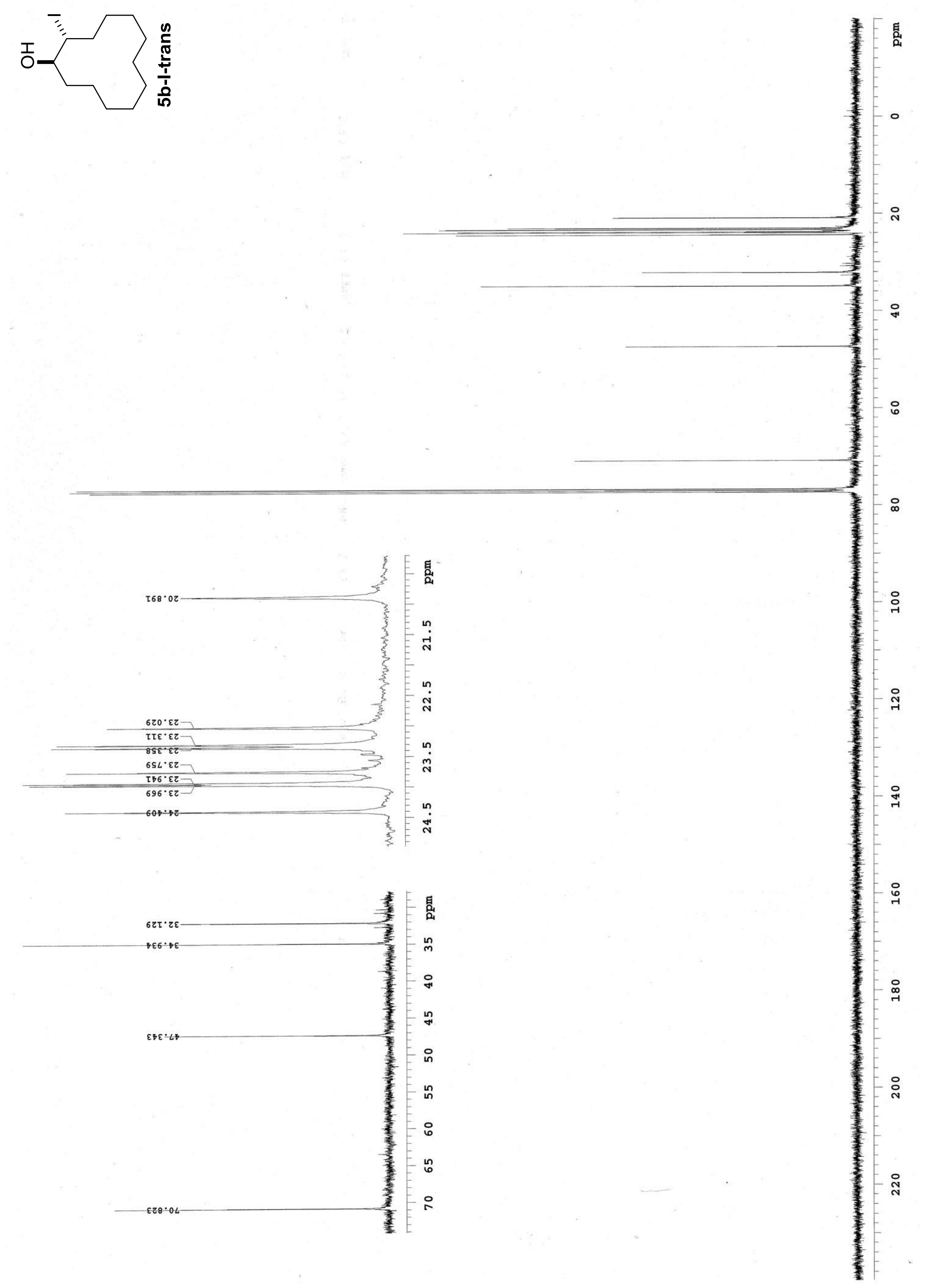
OH
I
5b-I-trans
20.891
23.029
23.311
23.358
23.759
23.941
23.969
24.409
32.129
34.934
47.343
70.823
ppm

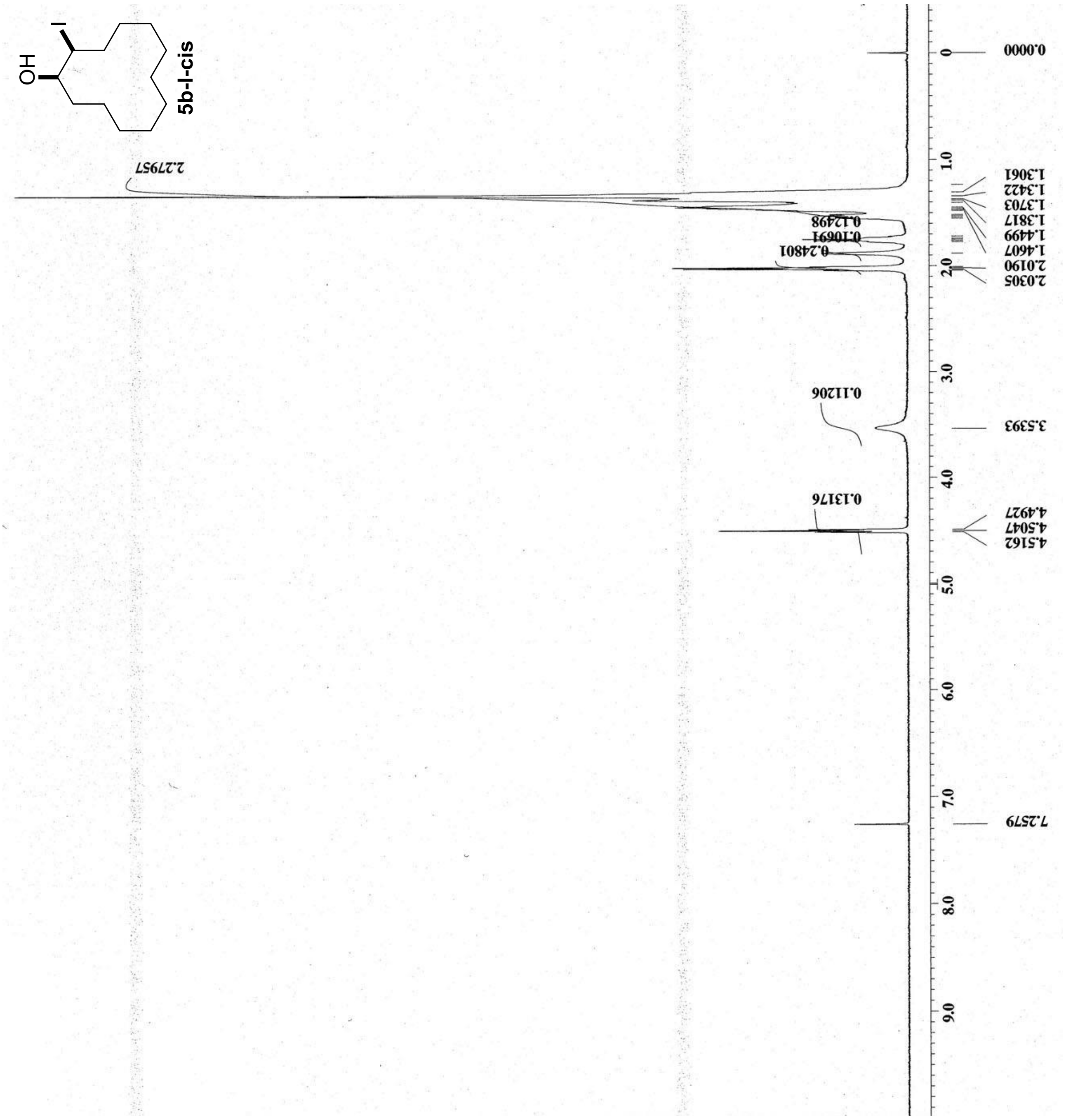
OH
I
5b-I-cis
0.0000
1.3061
1.3422
1.3703
1.3817
1.4499
1.4607
2.0190
2.0305
3.5393
4.4927
4.5047
4.5162
7.2579
2.27957
0.12498
0.10691
0.24801
0.11206
0.13176

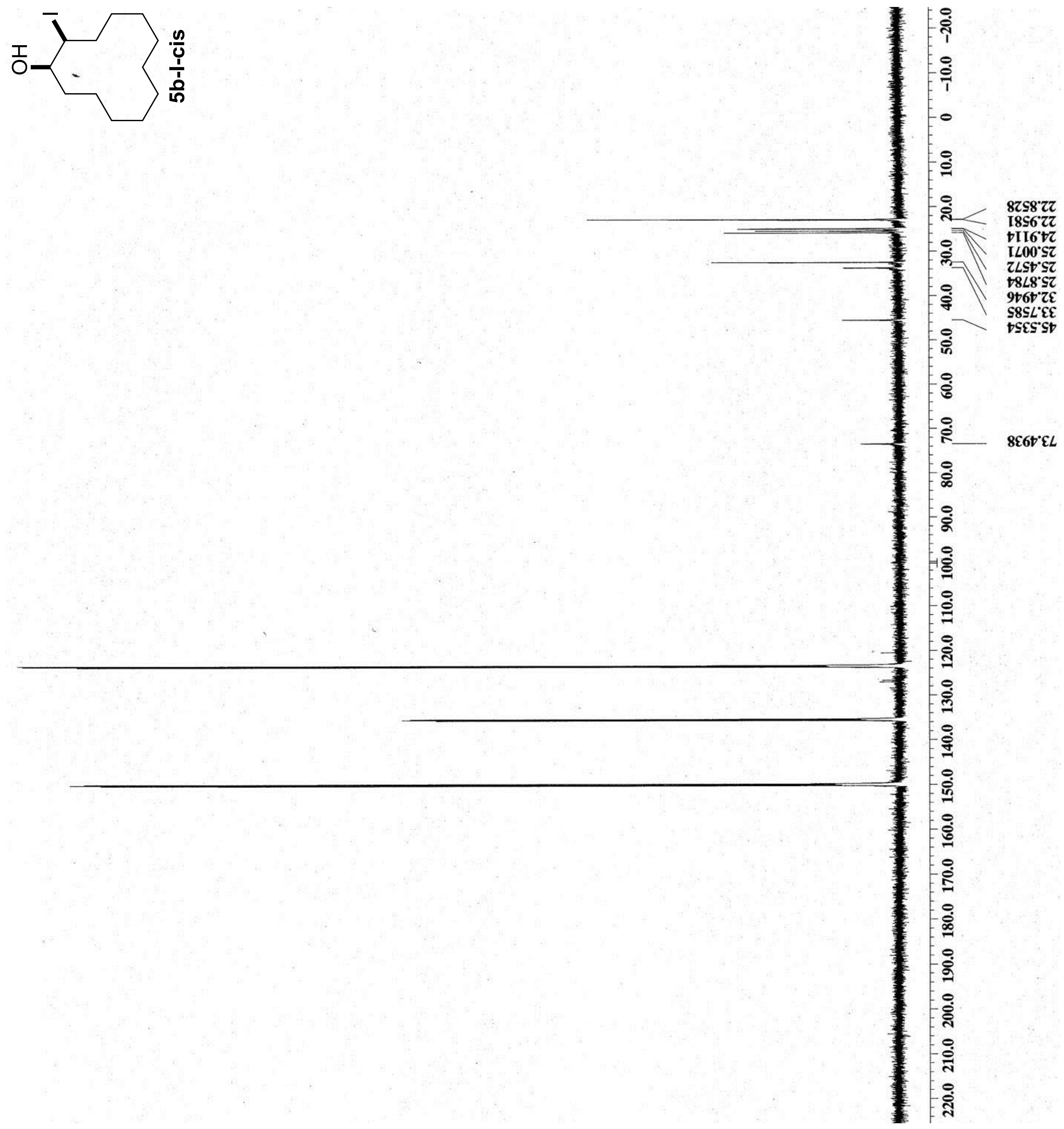
5b-I-cis
22.8528
22.9581
24.9114
25.0071
25.4572
25.8784
32.4946
33.7585
45.5354
73.4938

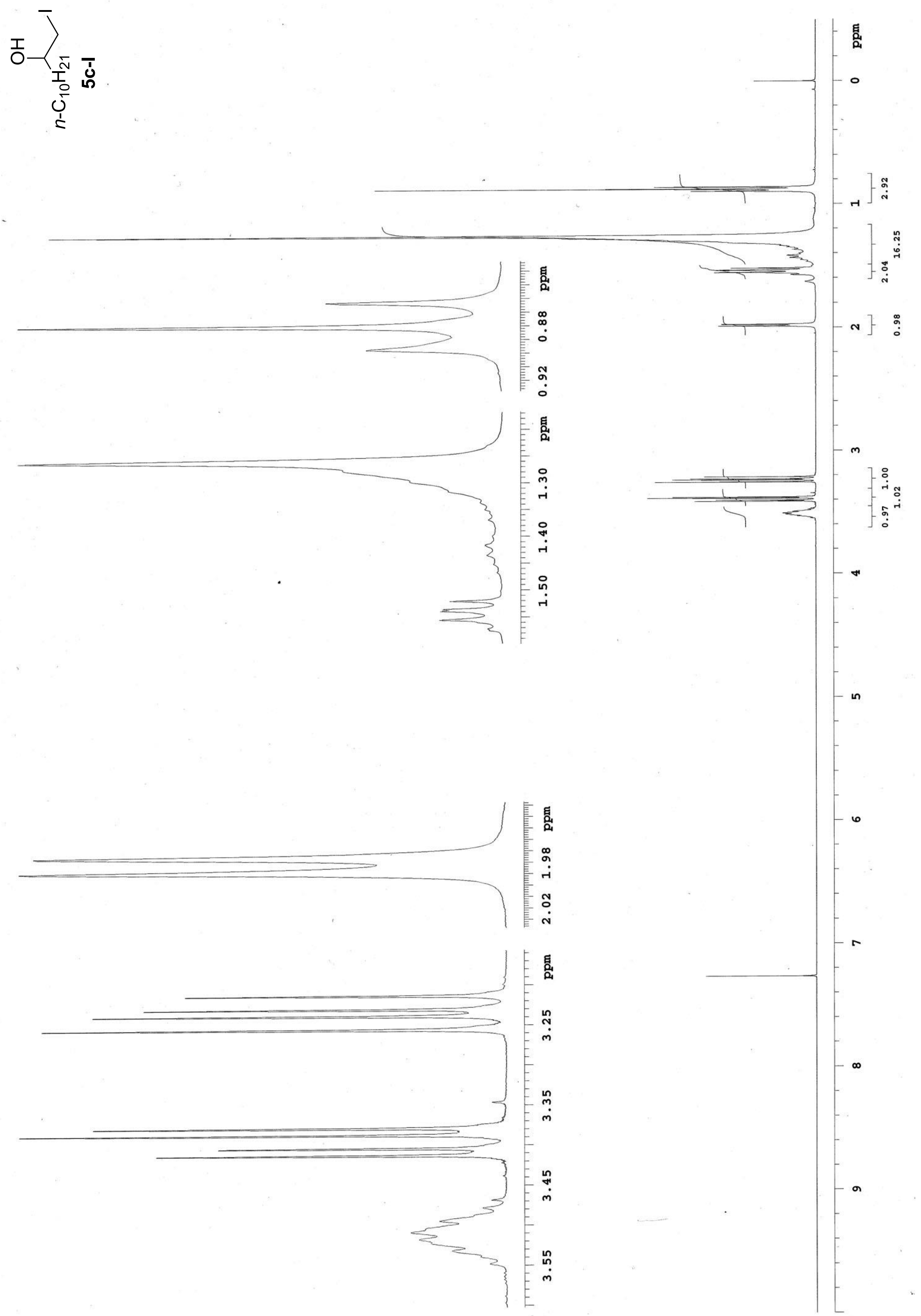
5c-I

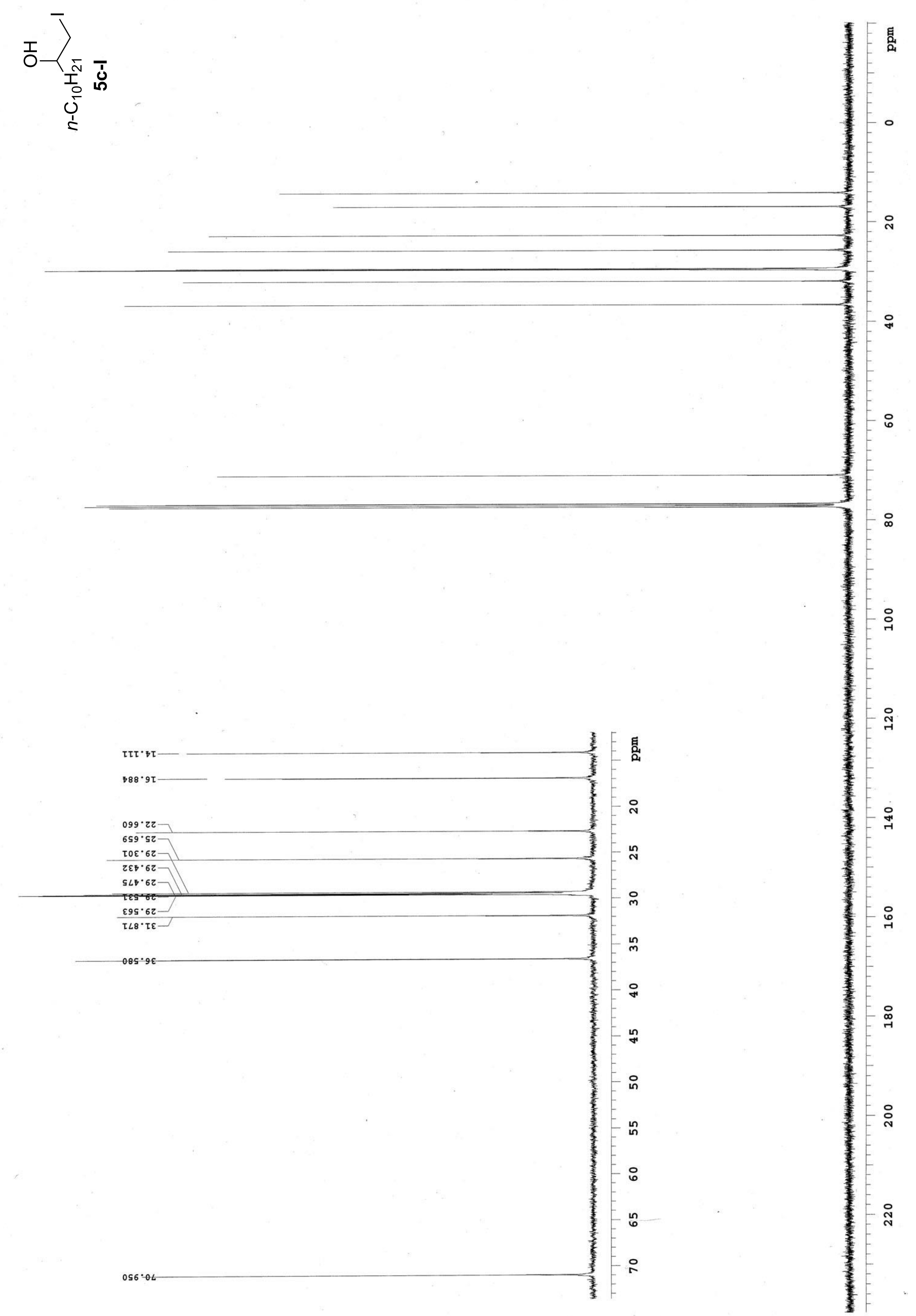
OH
n-C10H21
I
14.111
16.884
22.660
25.659
29.301
29.432
29.475
29.531
29.563
31.871
36.580
70.950
ppm
ppm

5c-I

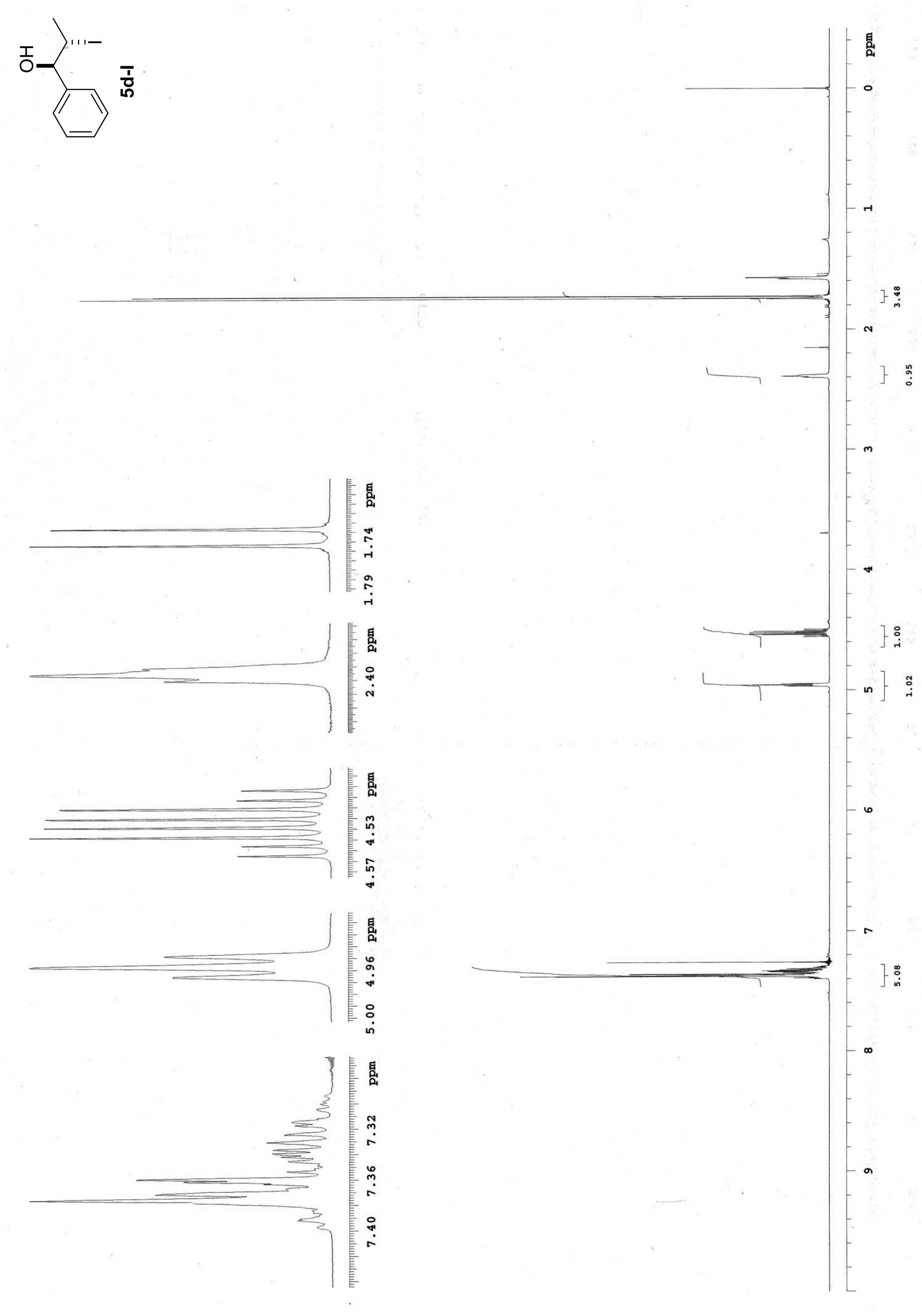

OH
5d-I
ppm
0
1
2
3
4
5
6
7
8
9
3.48
0.95
1.00
1.02
5.08
1.79 1.74 ppm
2.40 ppm
4.57 4.53 ppm
5.00 4.96 ppm
7.40 7.36 7.32 ppm

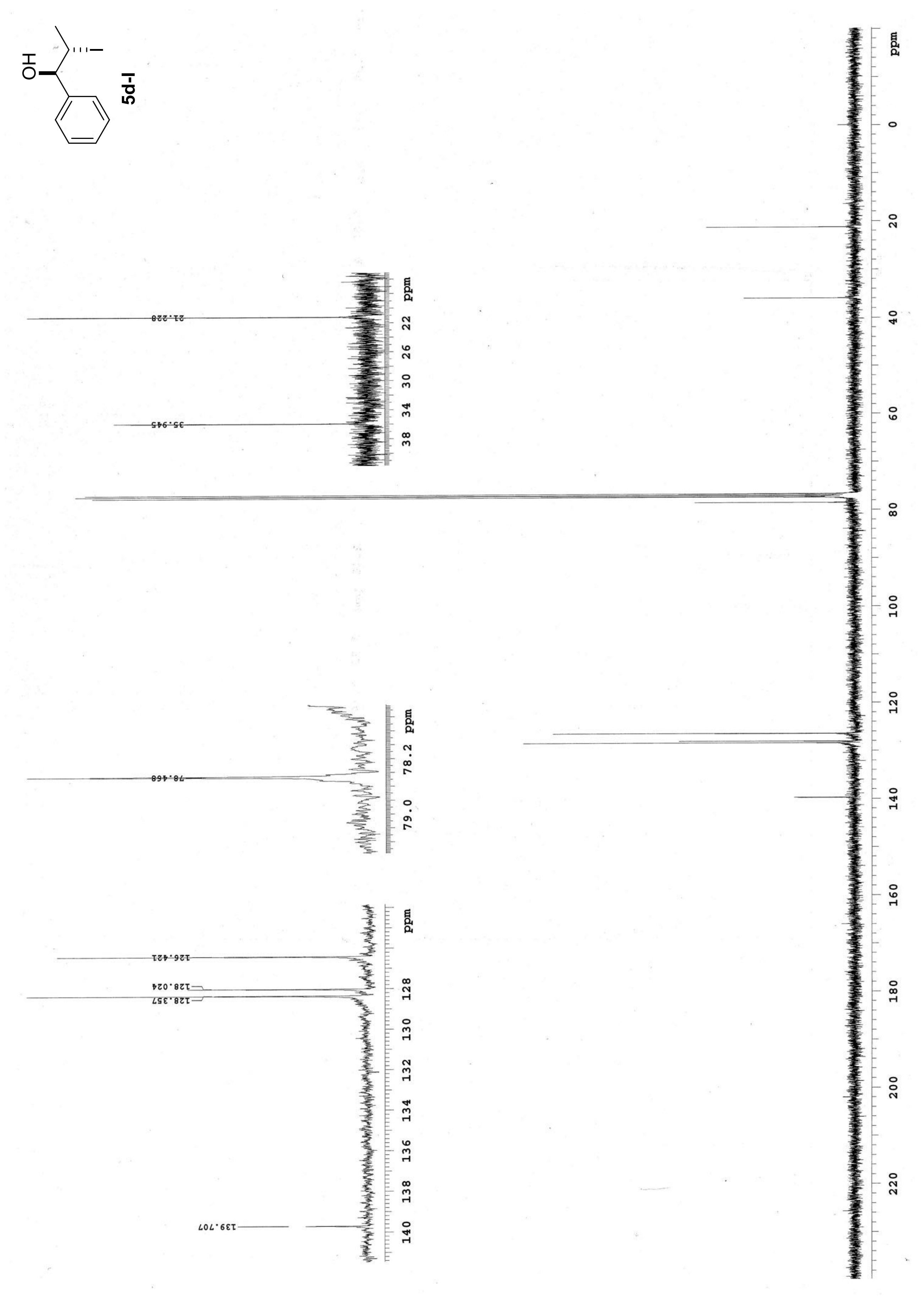
OH
5d-I
21.228
35.945
78.468
126.421
128.024
128.357
139.707
ppm

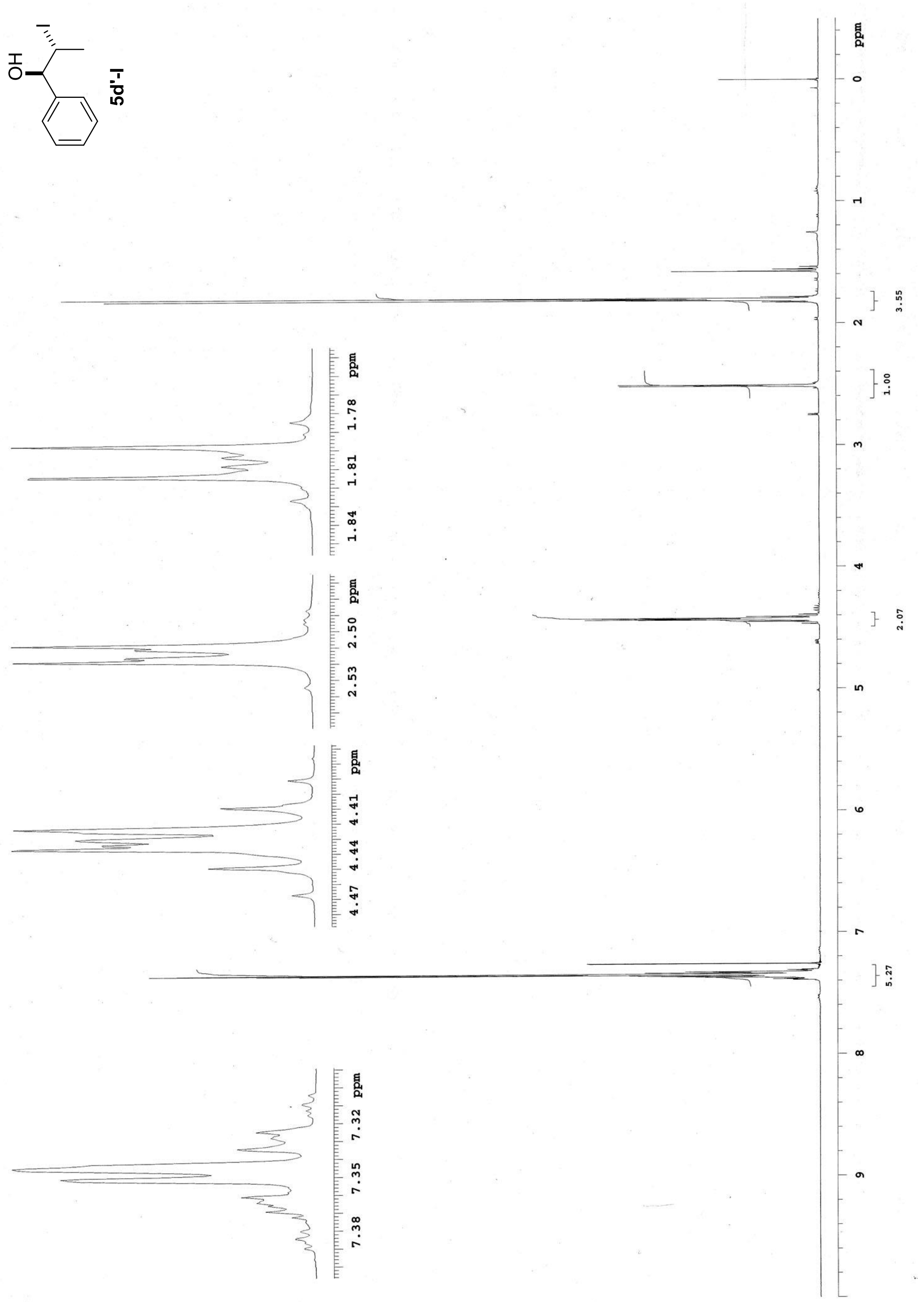
OH
I
5d'-I
ppm
3.55
1.00
2.07
5.27
7.38 7.35 7.32 ppm
4.47 4.44 4.41 ppm
2.53 2.50 ppm
1.84 1.81 1.78 ppm

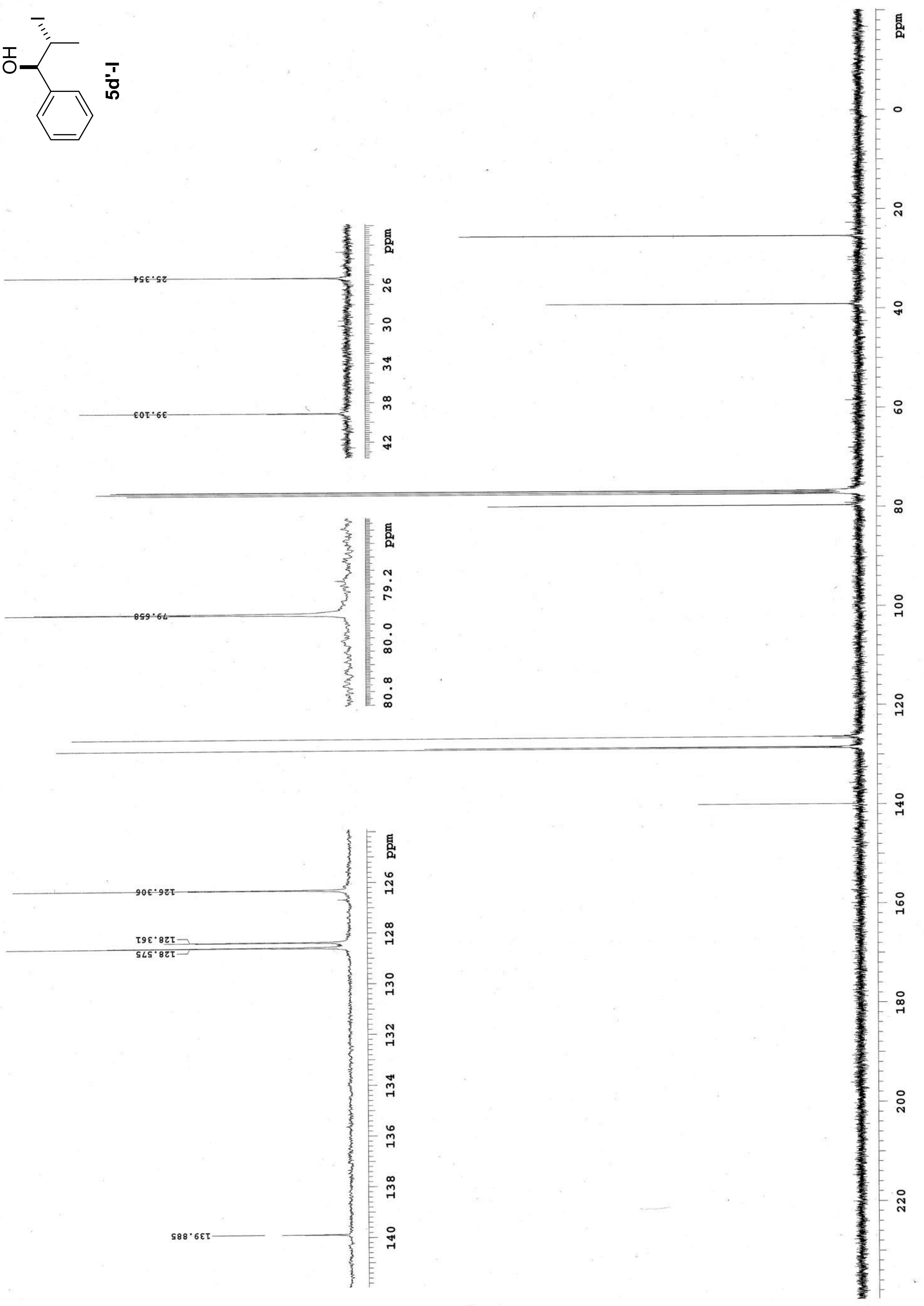
5d'-I
OH
I
25.354
39.103
79.658
126.306
128.361
128.575
139.885
ppm

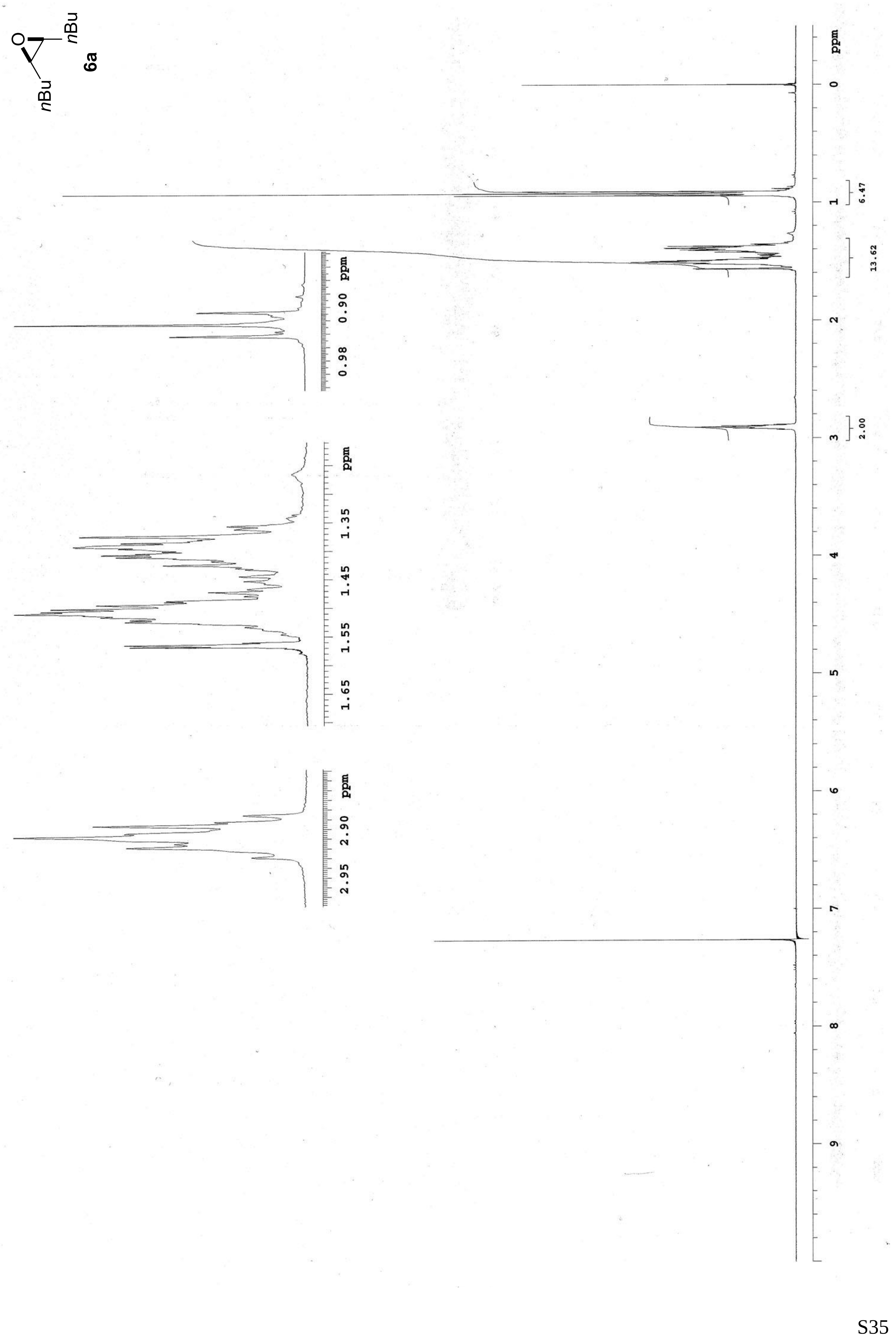
nBu
nBu
6a
ppm
0.90
0.98
1.35
1.45
1.55
1.65
2.90
2.95
0
1
2
3
4
5
6
7
8
9
6.47
13.62
2.00

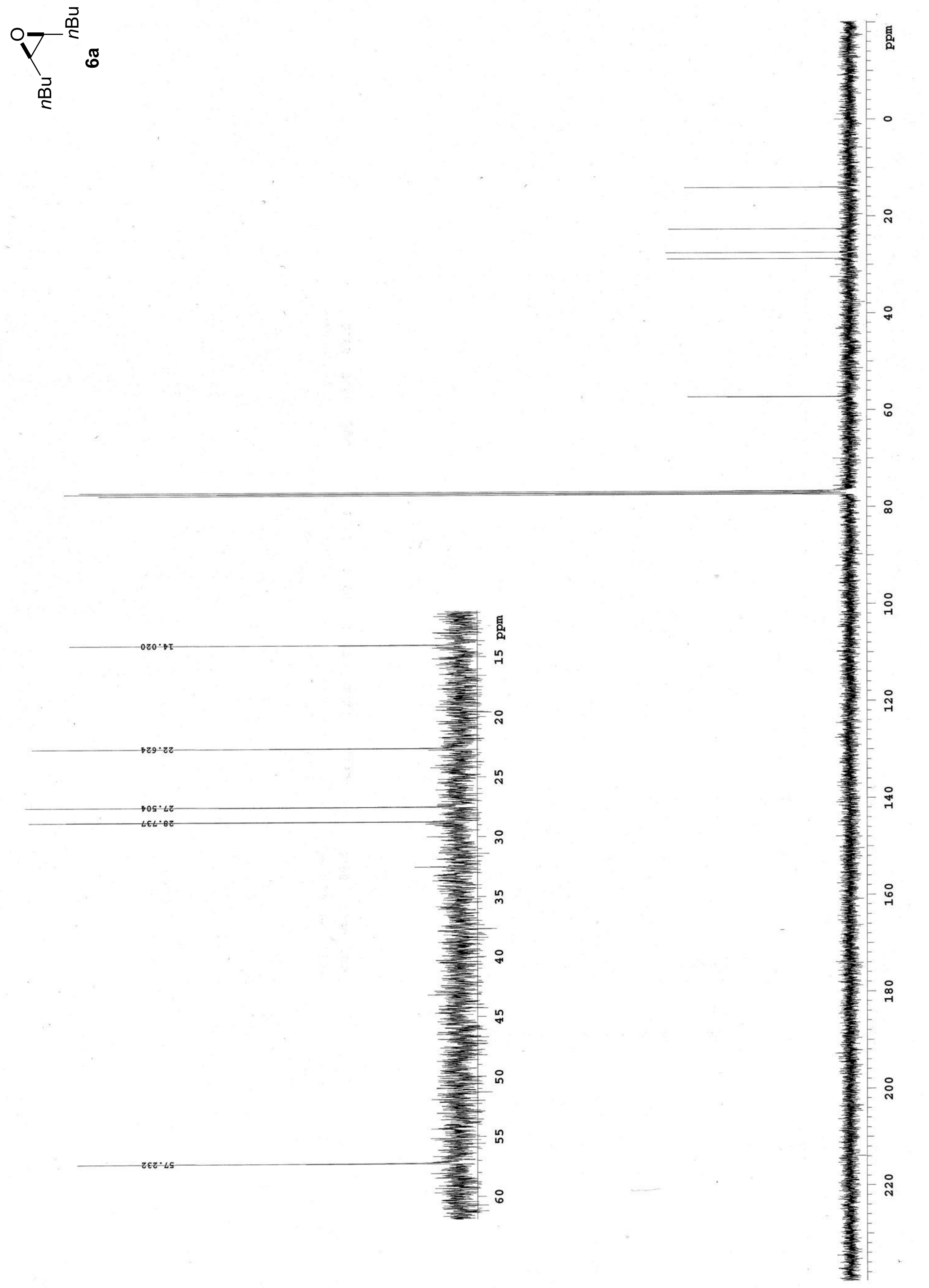
nBu
O
nBu
6a
14.020
22.624
27.504
28.737
57.232
ppm
15
20
25
30
35
40
45
50
55
60
ppm
0
20
40
60
80
100
120
140
160
180
200
220

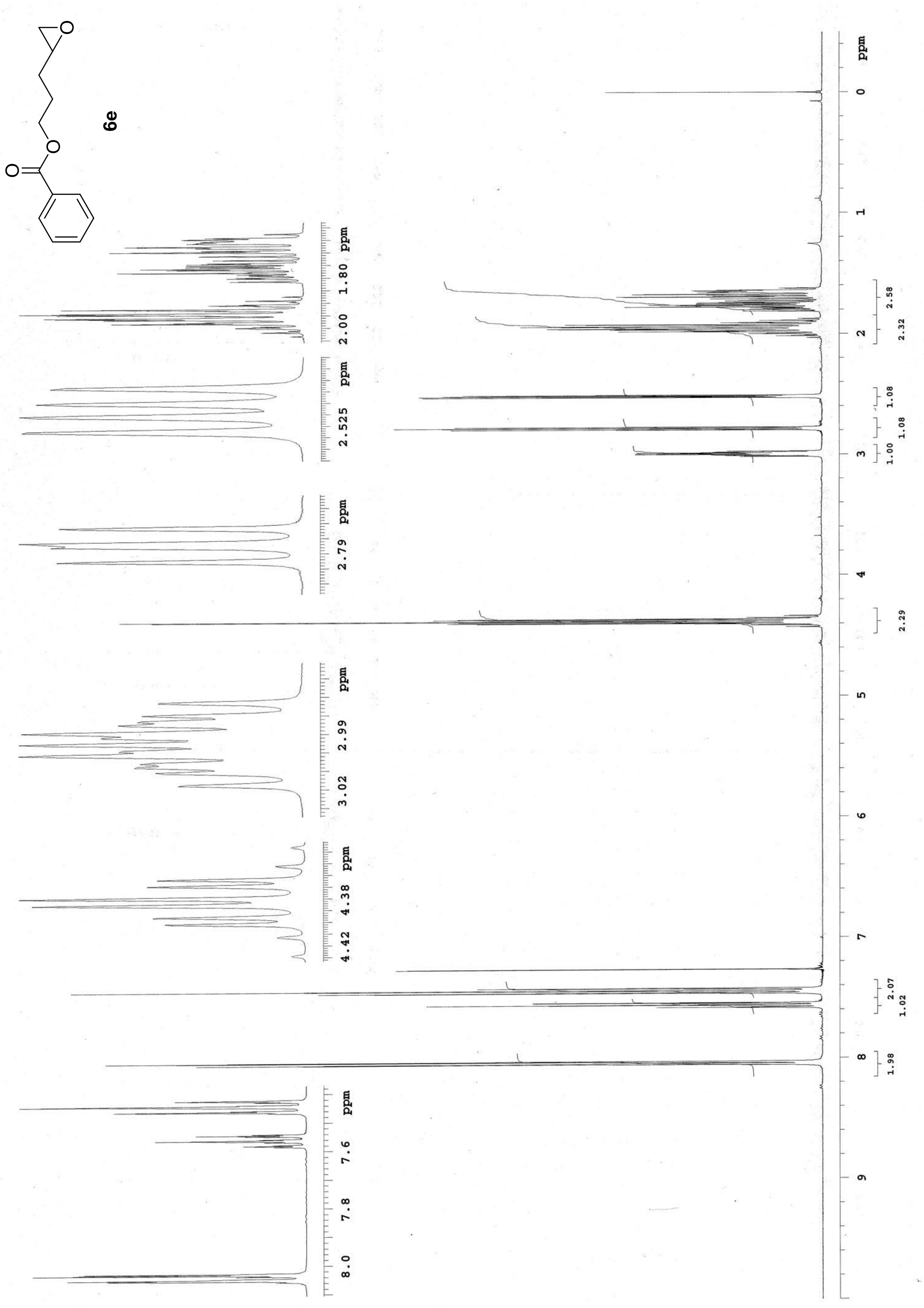

6e

6e

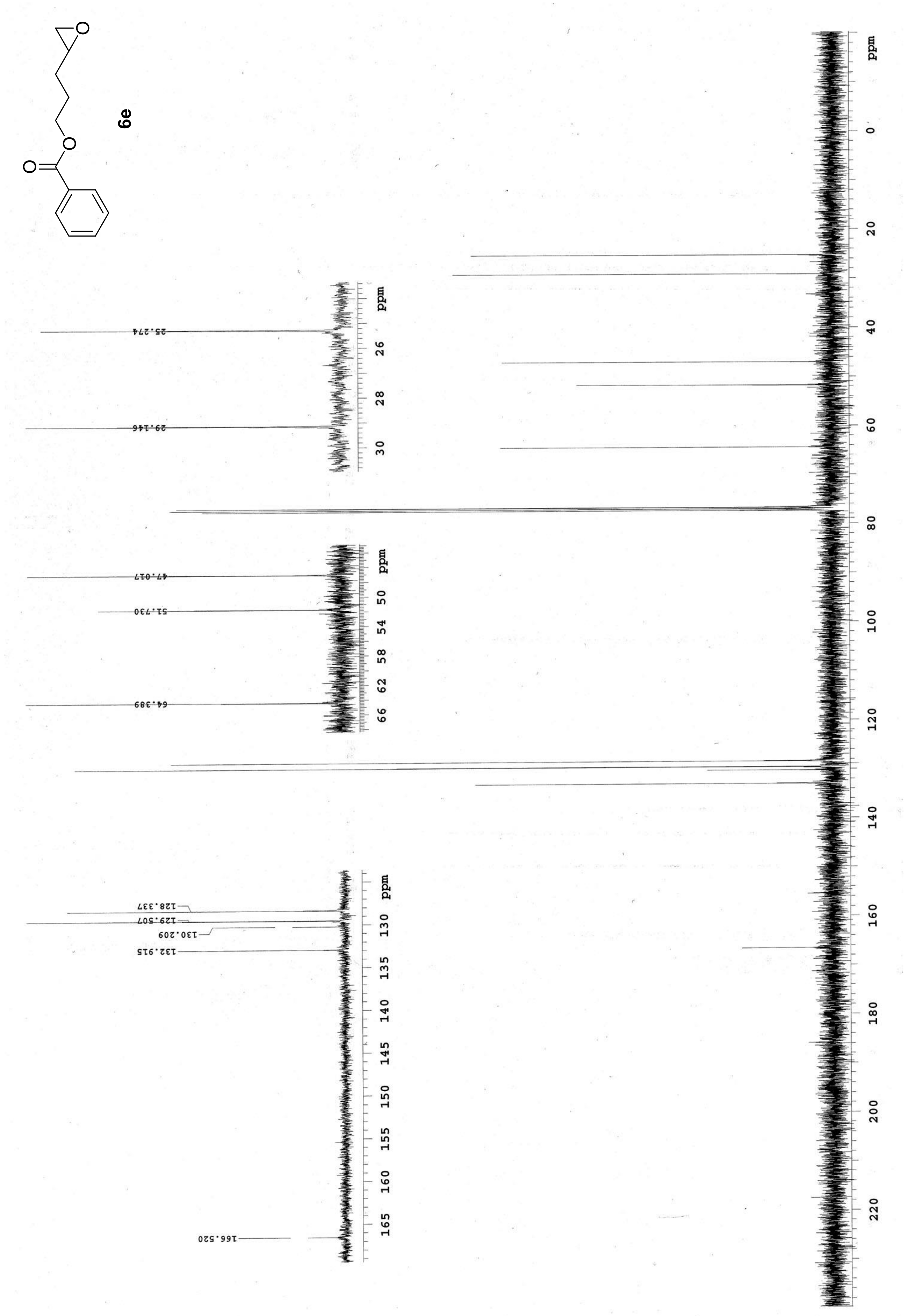

25.274
29.146
47.017
51.730
64.389
128.337
129.507
130.209
132.915
166.520
ppm

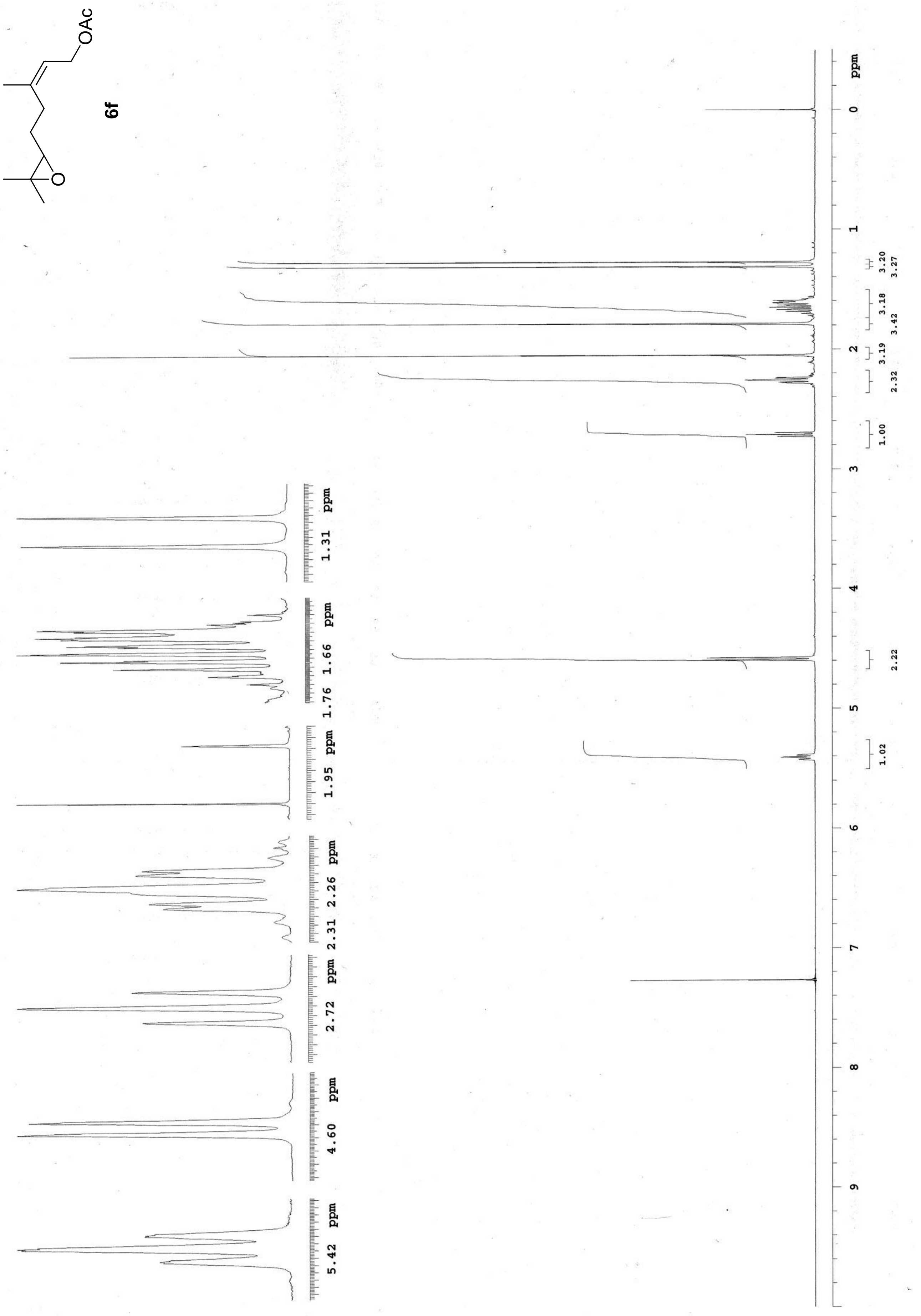
OAc
6f

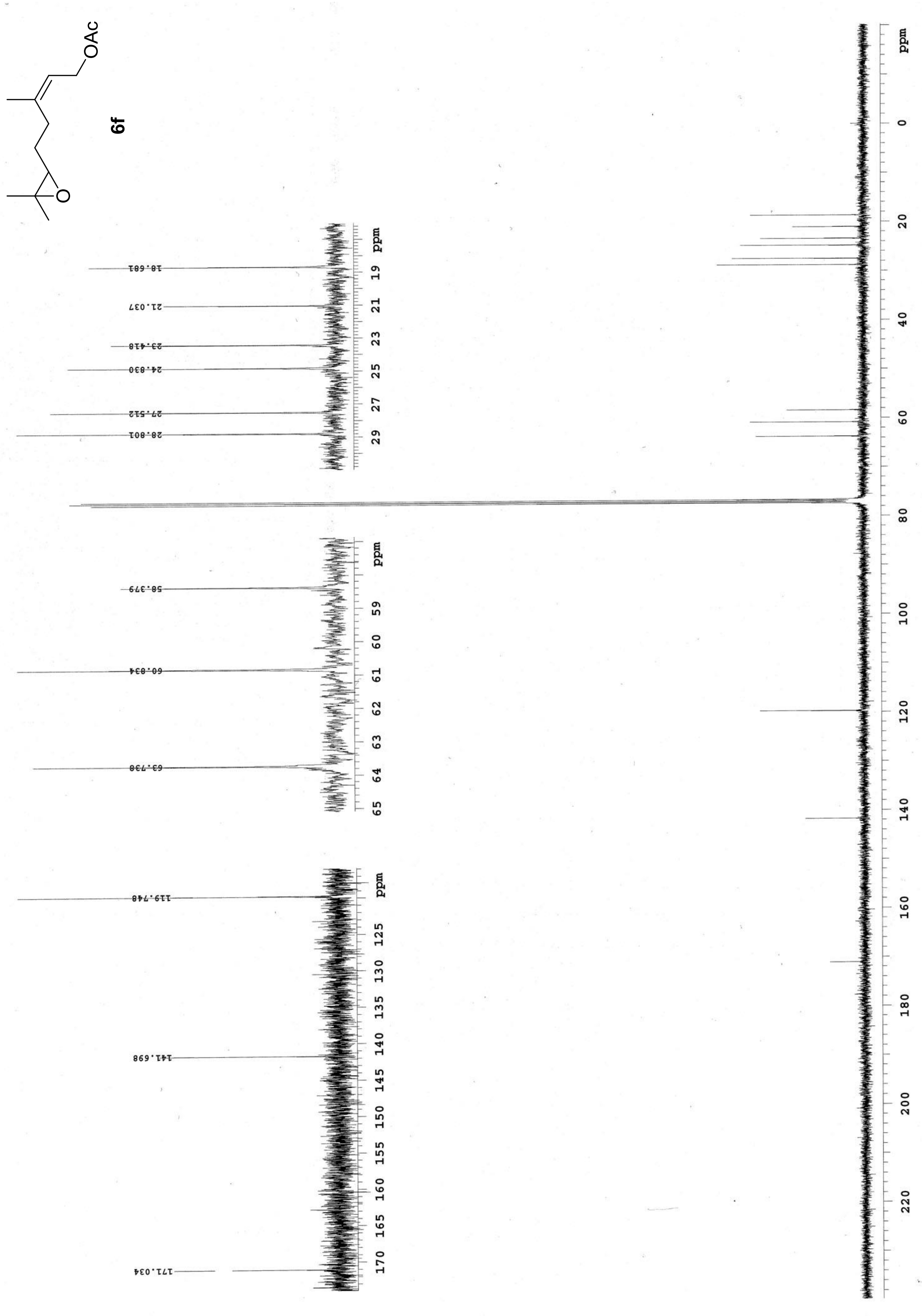
OAc
6f
18.681
21.037
23.418
24.830
27.512
28.801
58.379
60.834
63.738
119.748
141.698
171.034
ppm

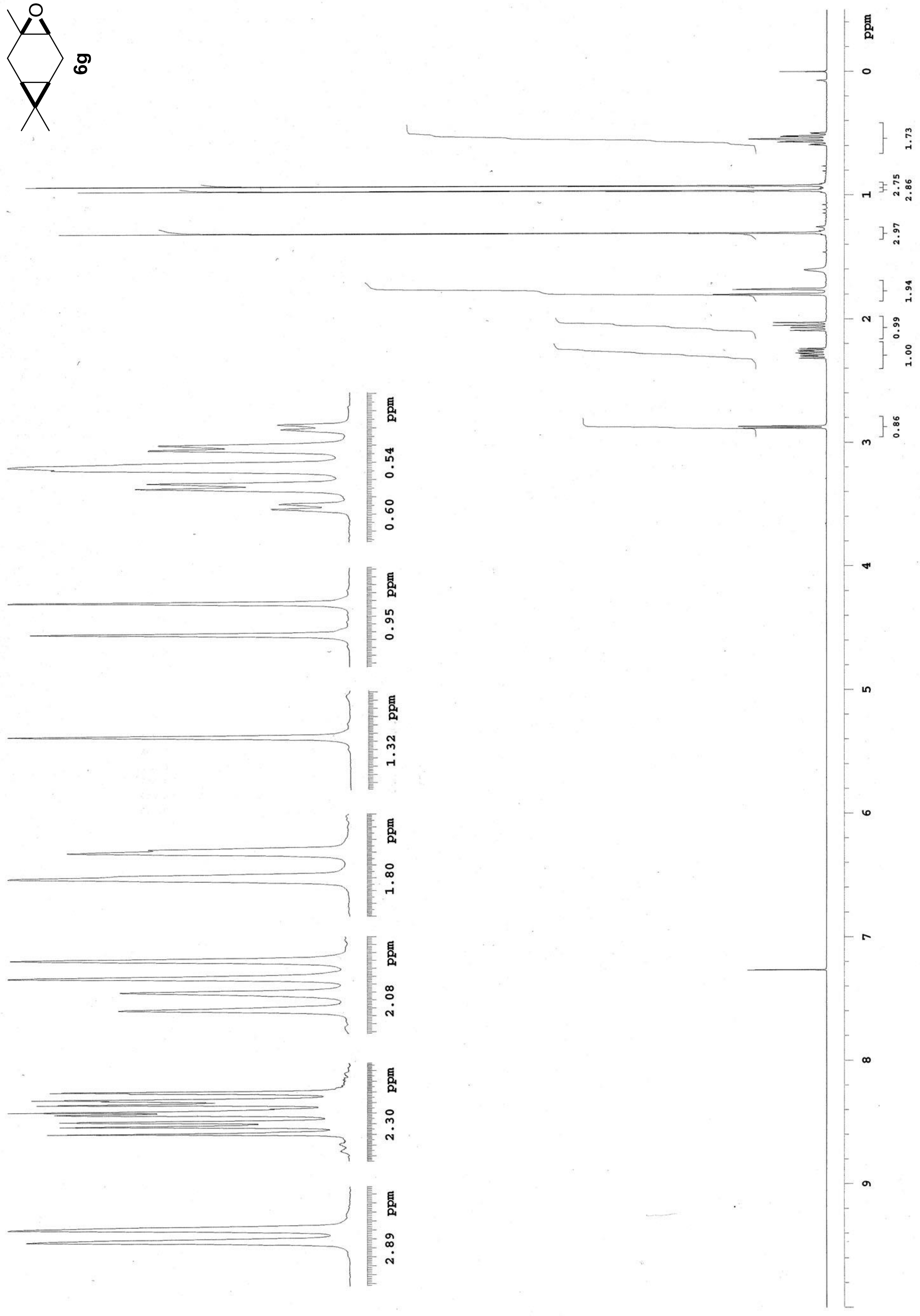
6g
ppm

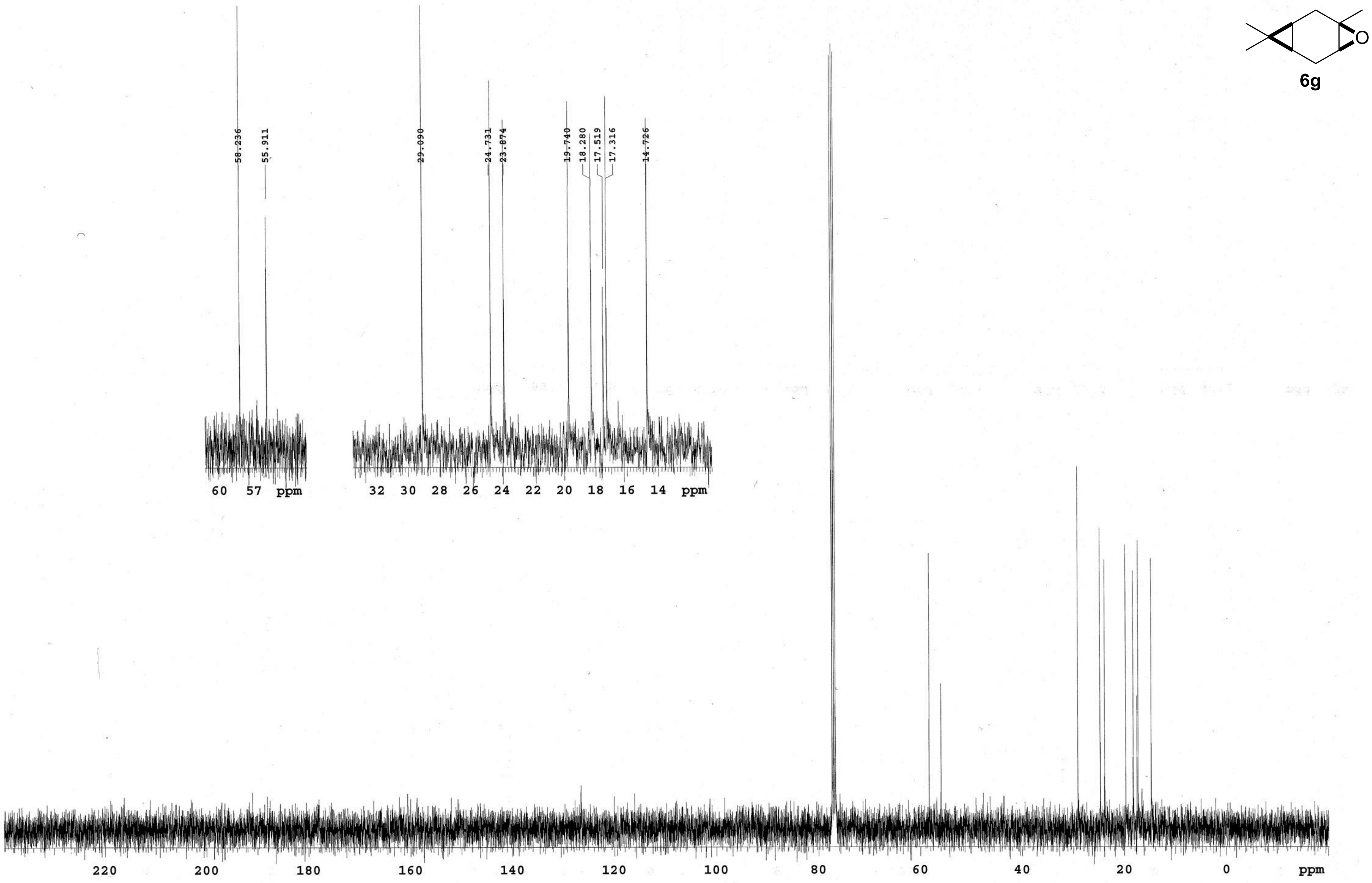
58.236
55.911
29.090
24.731
23.874
19.740
18.280
17.519
17.316
14.726
60 57 ppm
32 30 28 26 24 22 20 18 16 14 ppm
220 200 180 160 140 120 100 80 60 40 20 0 ppm

6g

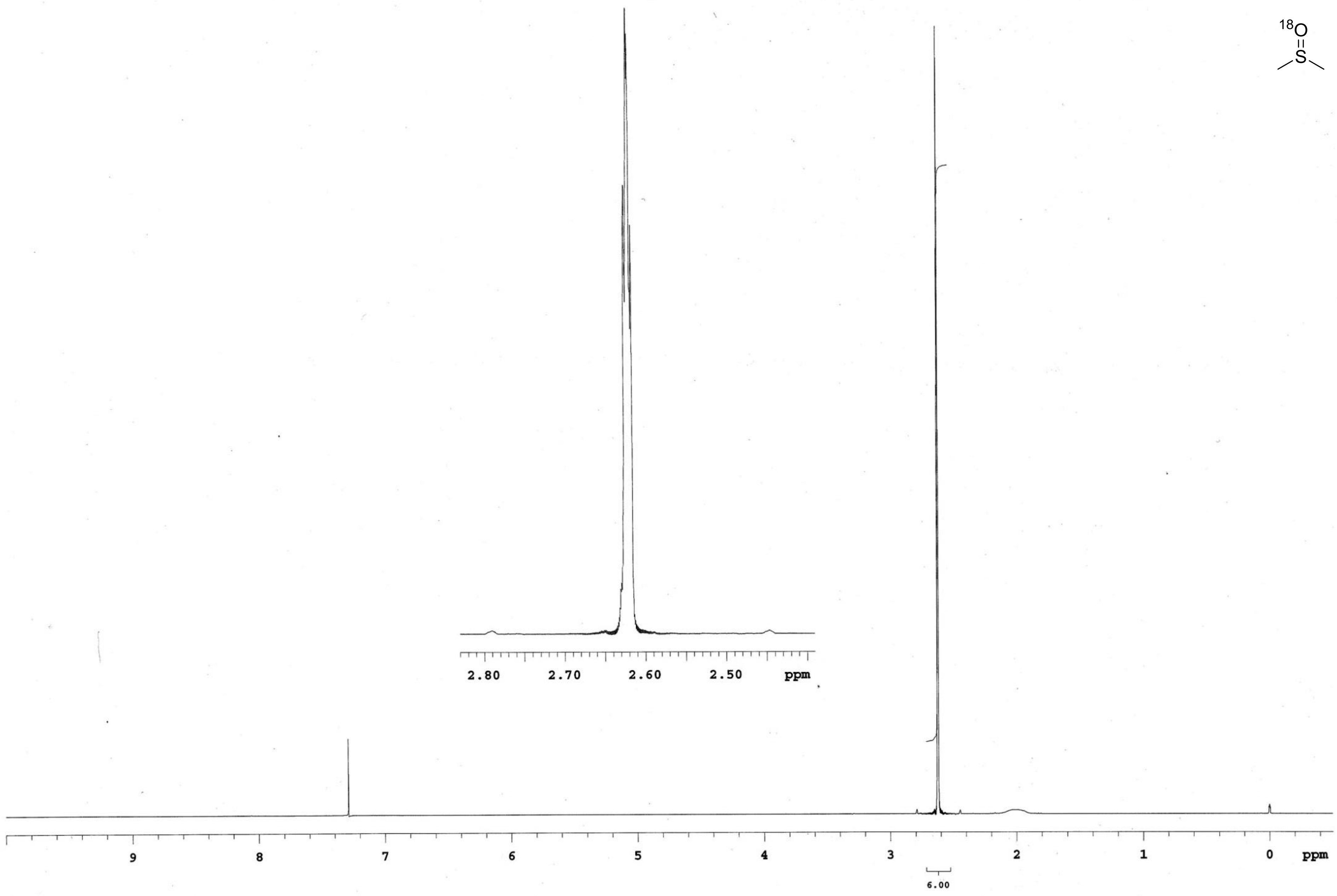
18O
S
2.80
2.70
2.60
2.50
ppm
9
8
7
6
5
4
3
2
1
0
ppm
6.00

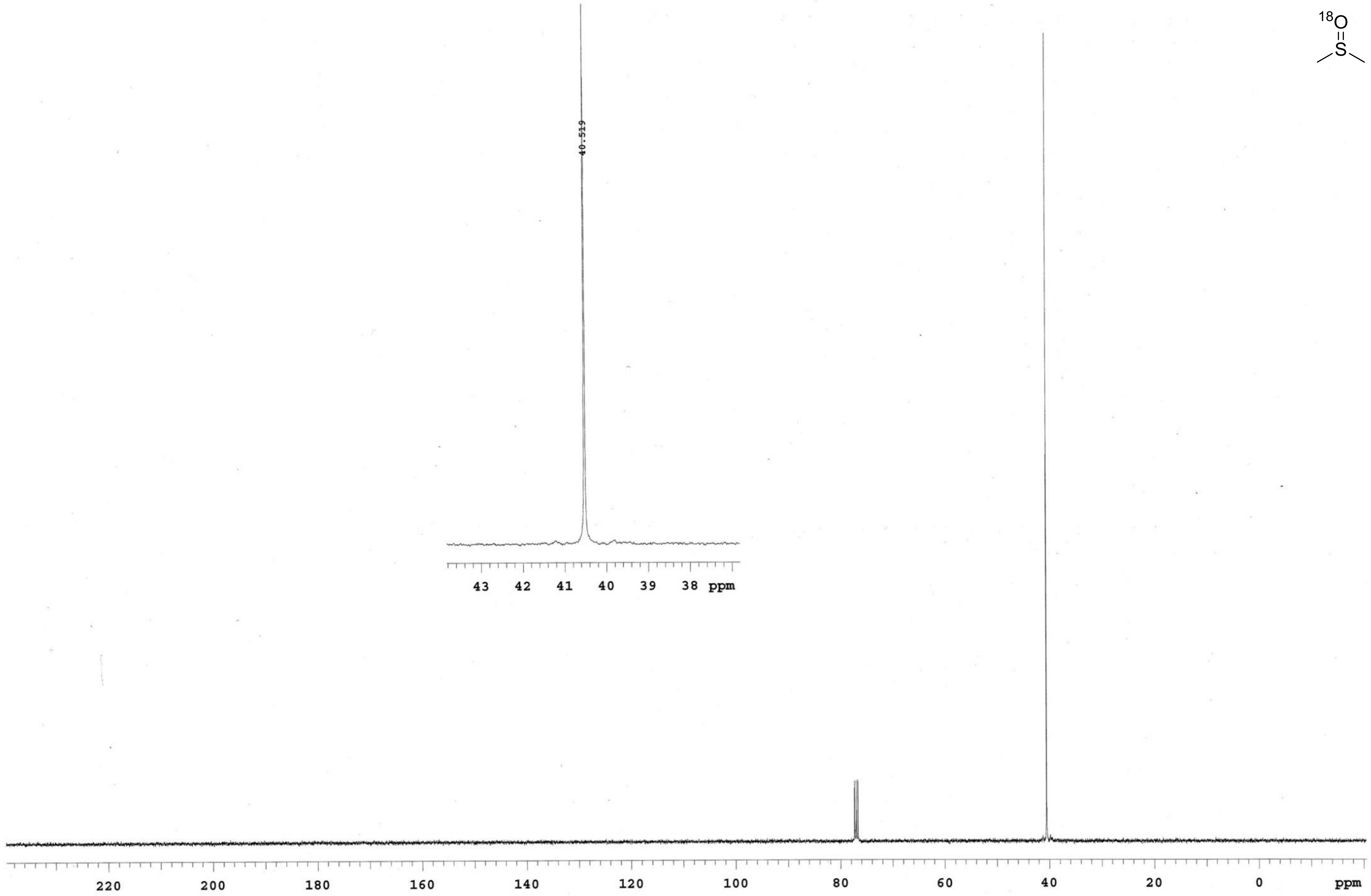
40.519
43
42
41
40
39
38
ppm
220
200
180
160
140
120
100
80
60
40
20
0
ppm

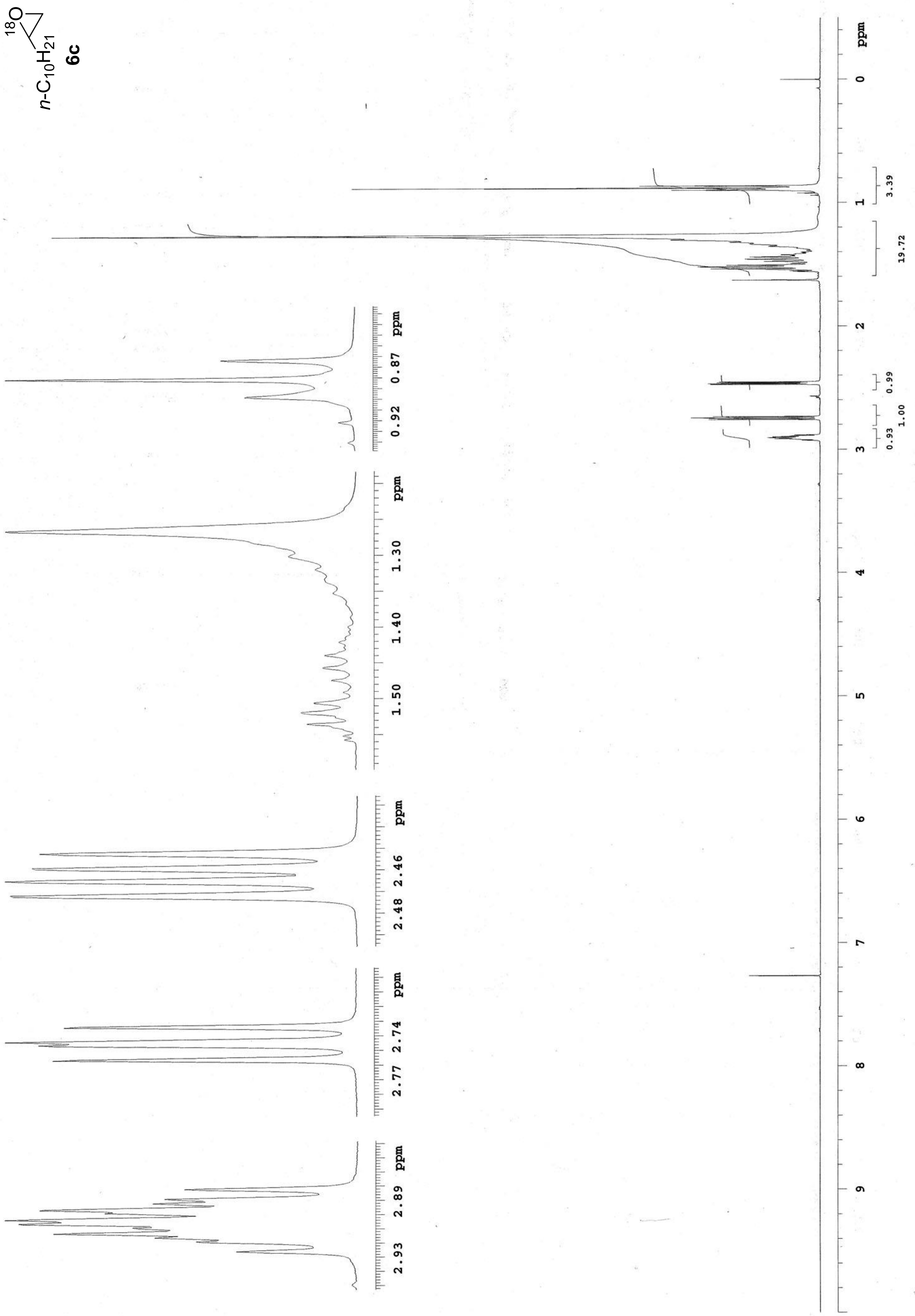

18O
n-C10H21
6c
ppm
0
1
2
3
4
5
6
7
8
9
3.39
19.72
0.99
1.00
0.93
0.92 0.87
1.50 1.40 1.30
2.48 2.46
2.77 2.74
2.93 2.89

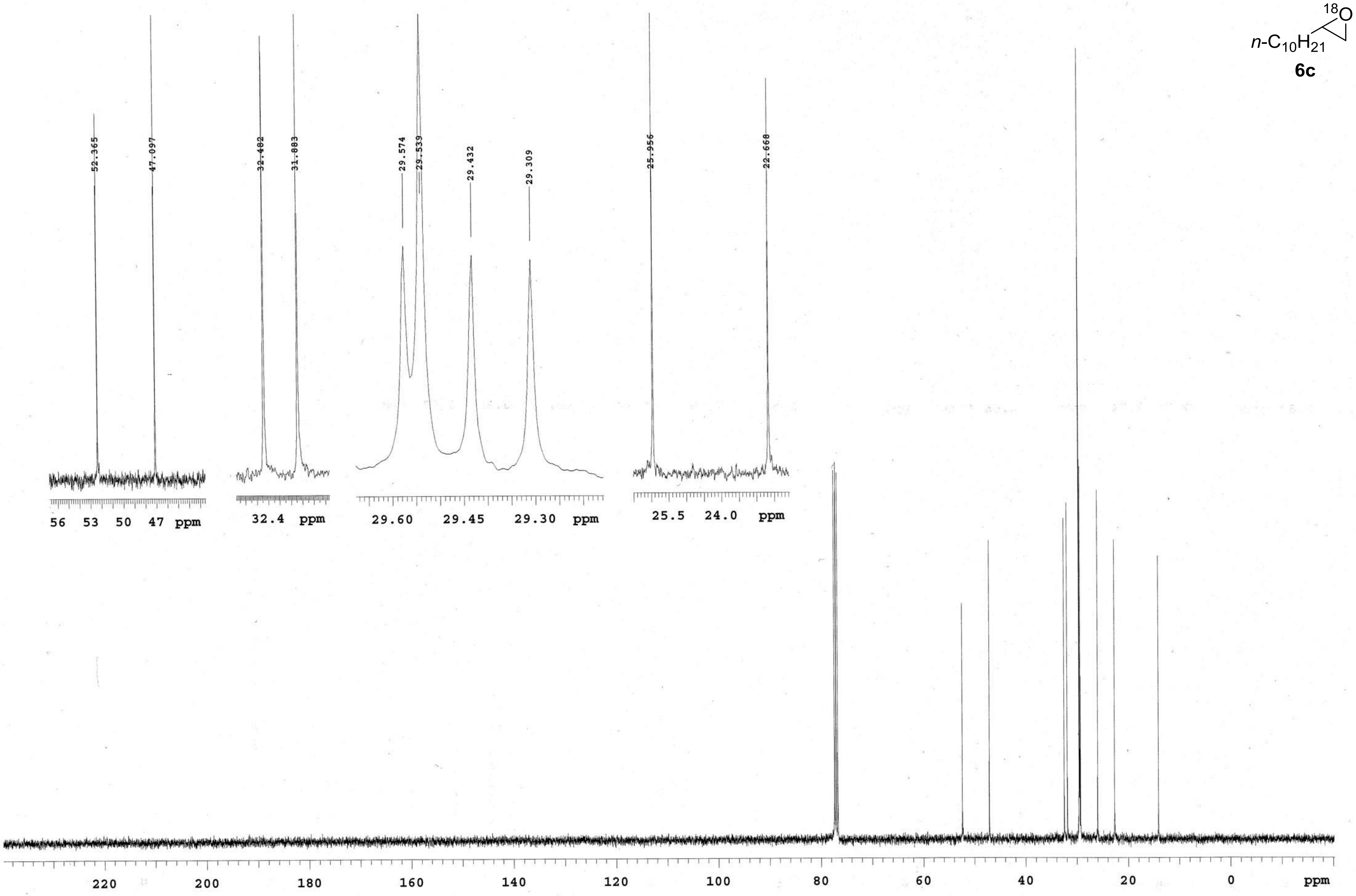
52.365
47.097
32.482
31.883
29.574
29.539
29.432
29.309
25.956
22.668
56 53 50 47 ppm
32.4 ppm
29.60 29.45 29.30 ppm
25.5 24.0 ppm
220 200 180 160 140 120 100 80 60 40 20 0 ppm

6c

8. Mass spectra of ^{18}O-labelled DMSO and 6c

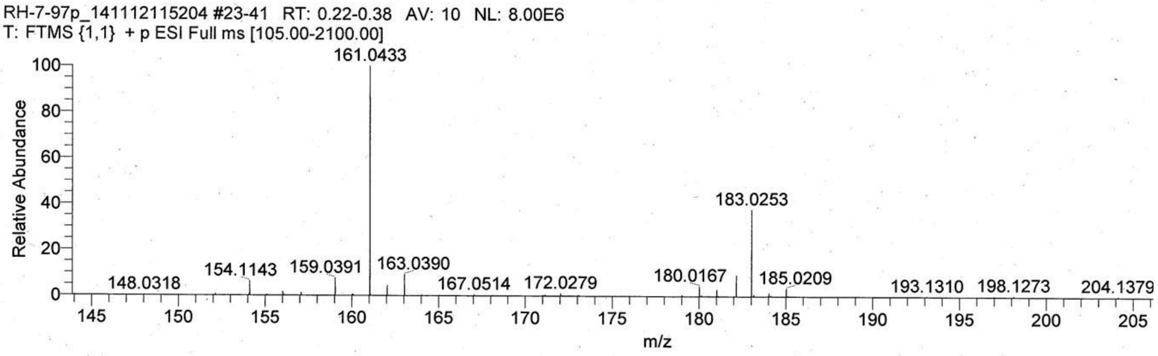

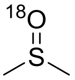

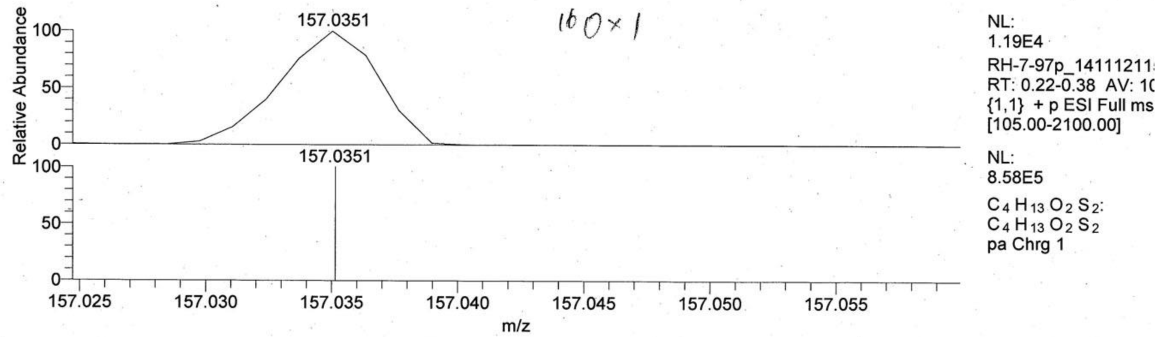

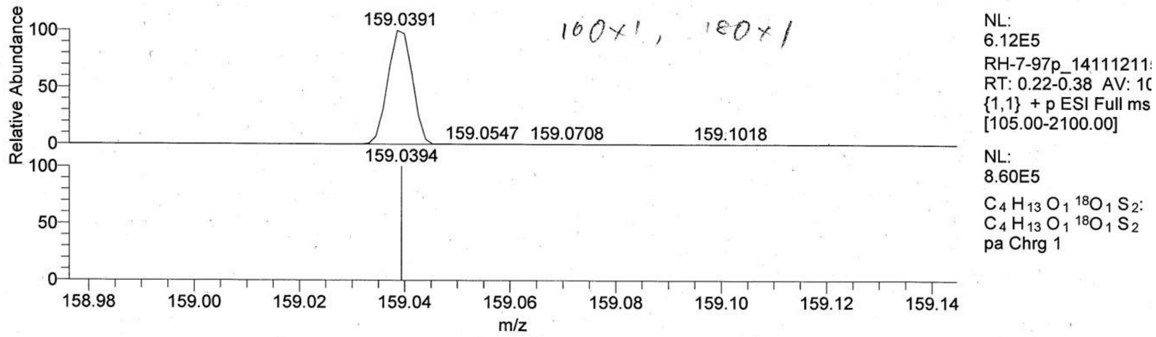

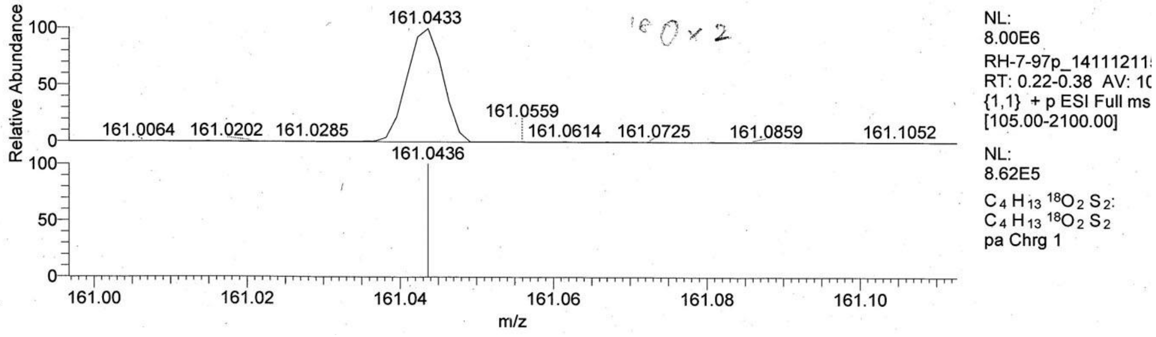

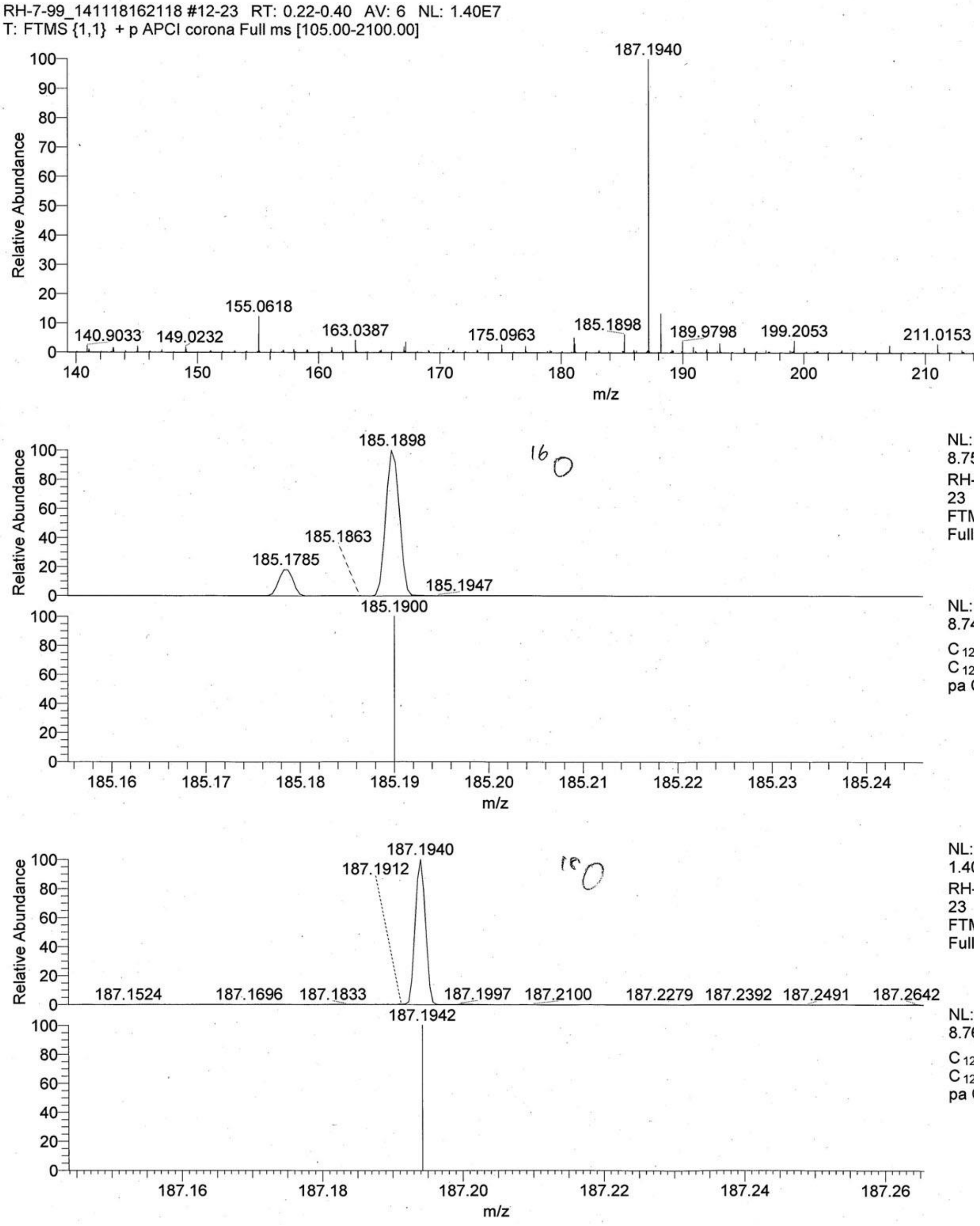
RH-7-99_141118162118 #12-23 RT: 0.22-0.40 AV: 6 NL: 1.40E7
T: FTMS {1,1} + p APCI corona Full ms [105.00-2100.00]
187.1940
155.0618
140.9033
149.0232
163.0387
175.0963
185.1898
189.9798
199.2053
211.0153
Relative Abundance
m/z
185.1898
16O
185.1863
185.1785
185.1947
185.1900
187.1940
187.1912
18O
187.1524
187.1696
187.1833
187.1997
187.2100
187.2279
187.2392
187.2491
187.2642
187.1942

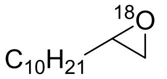
18O
C10H21

6c
